# Single cell analysis of cribriform prostate cancer reveals cell intrinsic and tumor microenvironmental pathways of aggressive disease

**DOI:** 10.1038/s41467-022-33780-1

**Published:** 2022-10-13

**Authors:** Hong Yuen Wong, Quanhu Sheng, Amanda B. Hesterberg, Sarah Croessmann, Brenda L. Rios, Khem Giri, Jorgen Jackson, Adam X. Miranda, Evan Watkins, Kerry R. Schaffer, Meredith Donahue, Elizabeth Winkler, David F. Penson, Joseph A. Smith, S. Duke Herrell, Amy N. Luckenbaugh, Daniel A. Barocas, Young J. Kim, Diana Graves, Giovanna A. Giannico, Jeffrey C. Rathmell, Ben H. Park, Jennifer B. Gordetsky, Paula J. Hurley

**Affiliations:** 1 Department of Medicine, Vanderbilt University Medical Center, Nashville, TN USA; 2 Department of Biostatistics, Vanderbilt University Medical Center, Nashville, TN USA; 3 Vanderbilt-Ingram Cancer Center, Nashville, TN USA; 4 Department of Urology, Vanderbilt University Medical Center, Nashville, TN USA; 5 Department of Otolaryngology-Head and Neck Surgery, Vanderbilt University Medical Center, Nashville, TN USA; 6 Regeneron Pharmaceuticals, Tarrytown, New York, USA; 7 Department of Pathology, Microbiology, and Immunology, Vanderbilt University Medical Center, Nashville, TN USA; 8 Vanderbilt Center for Immunobiology, Nashville, TN USA

**Keywords:** Prostate cancer, Cancer microenvironment, Tumour immunology

## Abstract

Cribriform prostate cancer, found in both invasive cribriform carcinoma (ICC) and intraductal carcinoma (IDC), is an aggressive histological subtype that is associated with progression to lethal disease. To delineate the molecular and cellular underpinnings of ICC/IDC aggressiveness, this study examines paired ICC/IDC and benign prostate surgical samples by single-cell RNA-sequencing, TCR sequencing, and histology. ICC/IDC cancer cells express genes associated with metastasis and targets with potential for therapeutic intervention. Pathway analyses and ligand/receptor status model cellular interactions among ICC/IDC and the tumor microenvironment (TME) including JAG1/NOTCH. The ICC/IDC TME is hallmarked by increased angiogenesis and immunosuppressive fibroblasts (*CTHRC1*^*+*^*ASPN*^*+*^*FAP*^*+*^*ENG*^*+*^) along with fewer T cells, elevated T cell dysfunction, and increased *C1QB*^*+*^*TREM2*^*+*^*APOE*^+^-M2 macrophages. These findings support that cancer cell intrinsic pathways and a complex immunosuppressive TME contribute to the aggressive phenotype of ICC/IDC. These data highlight potential therapeutic opportunities to restore immune signaling in patients with ICC/IDC that may afford better outcomes.

## Abstract (web summary)

The molecular and cellular underpinnings of cribriform prostate cancer aggressiveness remain to be explored. Here, the authors perform single-cell RNA-sequencing, TCR sequencing and histology and reveal cancer cell intrinsic pathways and an immunosuppressive tumour microenvironment.

## Introduction

Prostate cancer is one of the most common cancers in men in the US, and a leading cause of cancer-related death due to progression to metastatic disease. Outcomes for men with localized prostate cancer range from favorable to unfavorable. Although the majority of men enrolled in Active Surveillance programs or treated with curative-intent therapy for localized prostate cancer experience long-term metastatic progression-free survival, a portion of these men develop metastatic recurrence following therapy. Grade Groups^1–3^, a refined classification system based on the prior Gleason grading system^4^, is one of the strongest prognostic indicators of outcome for men with localized prostate cancer. Further stratification of risk classification by the presence or absence of cribriform morphology has been supported by several recent studies^5–11^. Current consensus guidelines^12,13^ classify cribriform morphology as well as glomeruloid, fused, and poorly formed as Gleason pattern 4^10^. Cribriform morphology is characterized by sheets of cells with intercellular lumina and can be found in both invasive cribriform carcinoma (ICC) as well as intraductal carcinoma (IDC)^10^. Interestingly, ICC and IDC frequently co-occur, with 47% of ICC intermixed with IDC and 68% of IDC intermixed with ICC^14^. Cribriform morphology is associated with adverse clinicopathologic findings and outcomes independent of Grade Group^5–11^. Men who have cribriform morphology detected at radical prostatectomy (RP) are more likely to experience biochemical recurrence^15^, metastatic recurrence^15^, and prostate cancer-specific death^16^ independent of Gleason score. The incidence of cribriform morphology has been reported to be present on 25–34% of prostate biopsies^17,18^. Collectively, these studies highlight the impact of ICC/IDC on a considerable portion of men with prostate cancer who therefore have an elevated risk of developing lethal prostate cancer. However, despite advancements in knowledge about worse clinical outcomes, currently there are no systemic therapies specified for this aggressive subtype.

Studies have begun to define genetic and molecular alterations associated with cribriform morphology in ICC and IDC. Analyses indicate that cribriform morphology is associated with increased genetic instability and copy number alterations^16,19,20^ as well as with genetic alterations in *ATM*, *SPOP, BRCA2, TP53, RB1*, and *PTEN*^16,19,21–24^. However, recent findings have identified PTEN loss to be associated with IDC but not ICC^25,26^. Key genetic alterations may impact MYC^16,19^, mTORC1^16^, MAPK^16^, KRAS^16^, JAK-STAT^16^, and EGFR^27^ pathway deregulation reported in cribriform prostate cancer. Epigenetic alterations may also contribute to pathway deregulation as cribriform morphology has been associated with increased EZH2 expression and elevated methylation^16^. EZH2 was recently shown to interact with *SCHLAP1*^28^, a long non-coding RNA associated with cribriform morphology^20,29^ and progression to metastasis^29–31^. While multiple genetic and molecular alterations have been associated with ICC/IDC, few are specific to this histological subtype, and causal mediators of ICC/IDC have yet to be definitively determined.

In contrast to tumor intrinsic alterations, limited studies have been reported on the tumor microenvironment (TME) associated with ICC/IDC. Several studies have posited that cribriform glands have increased hypoxia due to their distinctive architecture of contiguous epithelial cells without intervening stroma. This architectural pattern suggests that most cribriform cancer cells do not directly interface with surrounding stroma and may thereby have limited access to surrounding vasculature^32^. In support of this, patients with ICC/IDC demonstrated increased levels of hypoxia, which may contribute to the genetic instability and poor outcomes associated with ICC/IDC^20^. However, vascular infiltration patterns and other stromal cells associated with ICC/IDC have not been robustly assessed. A recent study comparing cribriform morphology to other Gleason pattern 4 histologic subtypes, as well as lower Gleason patterns, showed that *FAP*^+^*ASPN*^+^ cancer-associated fibroblasts (CAF) were enriched in regions directly adjacent to cribriform foci^33^. FAP has immunosuppressive functions in the TME^34,35^, yet little has been reported on the immune microenvironment associated with prostate ICC/IDC.

In this work, we identify both cancer cell-intrinsic and microenvironmental factors that likely contribute to the aggressive nature of ICC/IDC. We isolate paired benign-enriched and ICC/IDC-enriched unfixed prostate tissue obtained from RP specimens for single-cell RNA-sequencing (scRNAseq), T cell receptor (TCR) sequencing, and histological analyses. Herein, we find that ICC/IDC cancer cell heterogeneity is most notably governed by individual patient gene expression as opposed to commonly altered oncogenic pathways, including prostate cancer drivers like ERG and PTEN. Notwithstanding, *SCHLAP1* is distinctly increased in ICC/IDC cancer cells compared to benign prostate and other Gleason patterns including Gleason patterns 4 non-ICC and 5. *JAG1* is similarly elevated in ICC/IDC and our data support a model of ICC/IDC JAG1 mediated activation of NOTCH in endothelial cells and smooth muscle cells (SMC). Our findings also model potential interactions between ICC/IDC and CAF and indicate CAF likely expand from *APOD*^+^ peri-epithelial fibroblast progenitors potentially due to PDGF and FGF expression by ICC/IDC. ICC/IDC CAF have increased expression of immunosuppressive genes, and an ICC/IDC CAF gene signature based on upregulated genes, *CTHRC1*, *ASPN*, *FAP*, and *ENG* (CAFÉ CAF), is associated with worse outcomes. Flow cytometry and TCR sequencing indicate that the ICC/IDC TME has decreased immune infiltration with a lower fraction of T cells, reduced T cell clonality, and elevated T cell exhaustion markers when compared to benign prostate. The ICC/IDC TME is additionally associated with decreased inflammatory phenotypes, as evidenced by increased *C1QB*^*+*^*TREM2*^*+*^*APOE*^+^ macrophages. This study describes ICC/IDC heterogeneity at the single-cell level and comprehensively analyzes the associated TME. The findings herein support that ICC/IDC have cell intrinsic pathway activation that promotes angiogenesis and fibroblast activation, and that ICC/IDC are associated with an altered TME that leads to immunosuppression, thereby, preventing effective immune responses. With the recent advancements in targeted and immunotherapies, these findings have potential therapeutic implications.

## Results

### Altered epithelial and microenvironmental cell types in prostate ICC/IDC

**Fig. 1:**
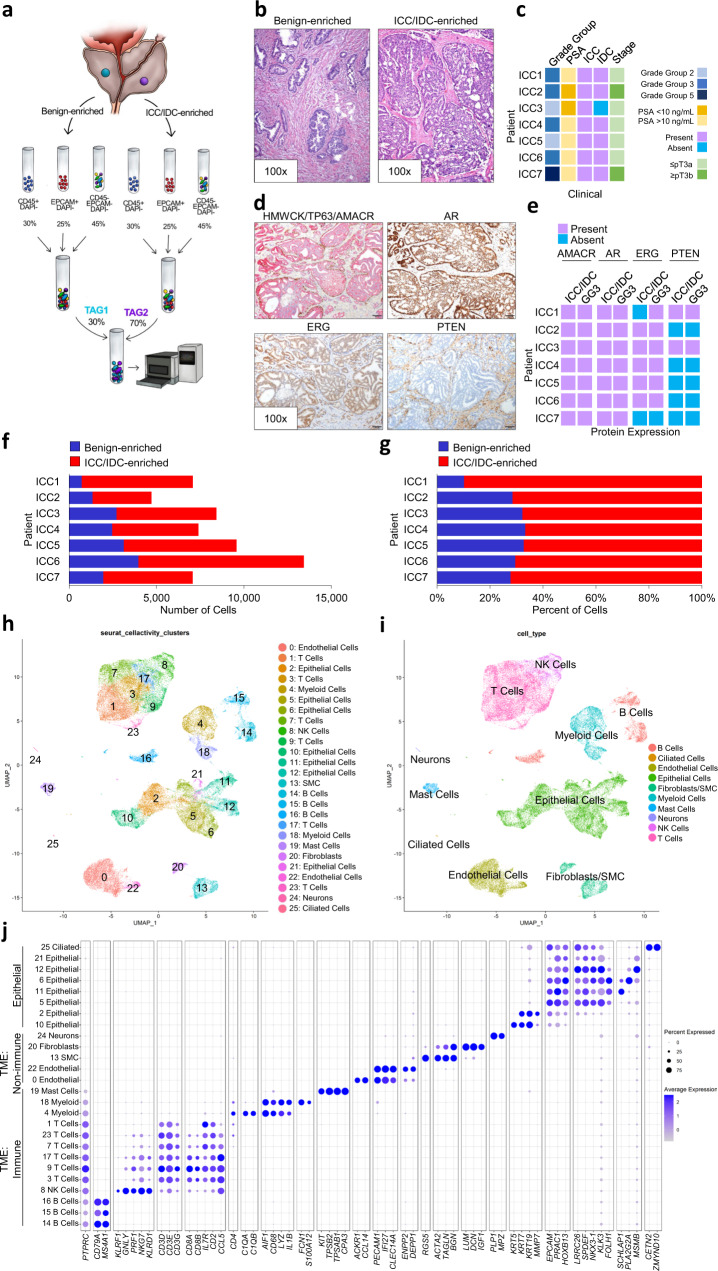
ScRNAseq of ICC/IDC-enriched and benign-enriched prostate. **a** Schematic of scRNAseq protocol of ICC/IDC-enriched and benign-enriched prostate. **b** Representative rapid frozen H&E of benign-enriched and ICC/IDC-enriched prostate isolated for scRNAseq at 100x, bar = 200 µm, (*n* = 7 biologically independent samples). This image and the six additional representative images are in Supplementary Fig. 1. **c** Patient clinical characteristics. **d** Representative HMWCK (High Molecular Weight Cytokeratin), TP63, AMACR, AR, ERG, and PTEN expression by IHC on patient FFPE prostate tissue from RP at 100x, bar = 100 µm, (*n* = 7 biologically independent samples). This image and the six additional representative images are in Supplementary Fig. 2a. **e** AMACR, AR, ERG, and PTEN expression by IHC per patient. **f**, **g** The number (**f**) and percent (**g**) of benign-enriched and ICC/IDC-enriched cells per patient. **h**, **i** Unsupervised graph-based clustering of all samples visualized by UMAP delineated by cluster (**h**) and cell-type (**i**). **j** Bubble plot of representative cell-type specific markers across all clusters. Source data are provided as a Source Data file.

To comprehensively analyze all cell types in ICC/IDC and the associated TME, paired benign-enriched and ICC/IDC-enriched prostate tissue from RP were isolated from 7 patients for scRNAseq (Fig. 1a, b). Benign-enriched and ICC/IDC-enriched regions were verified by obtaining a rapid frozen H&E of prostate tissue for histologic examination prior to processing (Fig. 1b and Supplementary Fig. 1). Overall, patients had Grade Group 2-5 prostate cancer that was either stage pT3aN0/X or pT3bN0 (Fig. 1c and Supplementary Table 1). FFPE sections from RP were examined by immunohistochemistry (IHC) for High Molecular Weight Cytokeratin (HMWCK), TP63, AMACR, AR, ERG, and PTEN (Fig. 1d, e and Supplementary Fig. 2a). In 6 patients, IDC was intermixed with ICC to varying proportions as determined by IHC staining for TP63 (Fig. 1d and Supplementary Fig. 2a). Cancer glands in all patients stained positive for AMACR (Fig. 1d, e and Supplementary Fig. 2a). Although ERG genomic rearrangements have not been previously associated with ICC/IDC^16^, ERG overexpression by IHC was detected in ICC/IDC from 5 patients (Fig. 1d, e and Supplementary Fig. 2a). Interestingly, ERG was overexpressed in Gleason pattern 3 from an additional patient but not in adjacent ICC/IDC. Consistent with prior findings^25,26^, homogenous PTEN loss by IHC was detected in 5 of the 6 patients with IDC (83%).

To facilitate analyses of all cell types found in the prostate, including cell types that were less abundant or difficult to isolate, cells were sorted for live cells and broad cell types (immune, epithelial, and other) by flow cytometry and then recombined for scRNAseq. Following tissue isolation and histological confirmation by H&E, paired benign-enriched and ICC/IDC-enriched samples were single-cell disassociated, stained with DAPI and antibodies against CD45 (pan-immune marker) and EpCAM (pan-epithelial marker), tagged for multi-plex sequencing, and flow-sorted into three DAPI^-^ (live) populations that were CD45^+^ (immune cells), EpCAM^+^ (epithelial cells), or CD45^-^EpCAM^-^ (cells other than immune and epithelial cells such as endothelial cells, smooth muscle cells, fibroblasts, and nerve cells). DAPI^-^ cells from benign-enriched and ICC/IDC-enriched tissue were then recombined at a ratio of 30% CD45^+^, 25% EpCAM^+^, and 45% CD45^-^EpCAM^-^ (Fig. 1a). Benign-enriched and ICC/IDC-enriched cells were then mixed at a 30:70 ratio, respectively, for scRNAseq and TCR VDJ sequencing using 10X genomics (Fig. 1a). After filtering low-quality cells and doublets, over 57,000 cells in total from 7 patients were analyzed by scRNAseq and over 15,000 of these cells were additionally analyzed by TCR sequencing (Fig. 1f, g and Supplementary Tables 2–5). The mean number of total cells analyzed per patient was 8242.

**Fig. 2:**
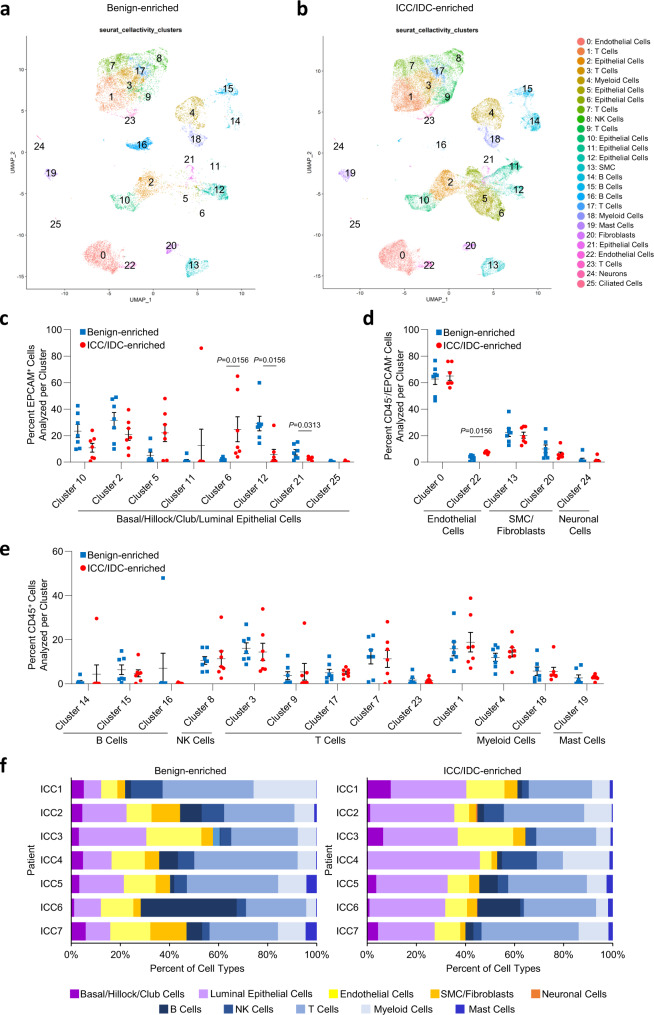
Epithelial and microenvironmental cell types were altered in prostate ICC/IDC. **a**, **b** Unsupervised graph-based clustering of all samples visualized by UMAP delineated by benign-enriched (**a**) and ICC/IDC-enriched (**b**) prostate. **c**–**e** Percent total of EpCAM^+^ (**c**), EpCAM^-^/CD45^-^ (**d**), and CD45^+^ (**e**) benign-enriched and ICC/IDC-enriched prostate cells per cluster. Graphs shown as mean ± SEM and analyzed by Wilcoxon matched-pair signed rank two-tailed test; *n* = 7 biologically independent samples. The partial graph (endothelial cells) in **d** is also shown in Fig. 4 (**g**). **f** Percent of cell type analyzed per patient delineated by benign-enriched and ICC/IDC-enriched prostate. Source data are provided as a Source Data file.

Unsupervised graph-based clustering and accompanying visualization with the Uniform Manifold Approximation and Projection (UMAP) algorithm yielded 26 clusters encompassing multiple cell types, including immune, endothelial, SMC, fibroblasts, and epithelial (Fig. 1h–j). Sample contribution to each cluster was variable, but most clusters were derived from a relatively even distribution of samples (Supplementary Fig. 3a, b, Supplementary Tables 6 and 7). Within the epithelial clusters, cluster 6 was significantly increased in ICC/IDC-enriched tumors compared to benign-enriched prostate, while clusters 12 and 21 were significantly decreased (Fig. 2a–f, and Supplementary Fig. 3b). Amongst the non-immune TME (CD45^-^/EpCAM^-^), cluster 22 (endothelial cells) was increased in ICC/IDC-enriched tumors compared to benign-enriched prostate (Fig. 2d).

### Increased *SCHLAP1* and *JAG1* in prostate ICC/IDC

**Fig. 3:**
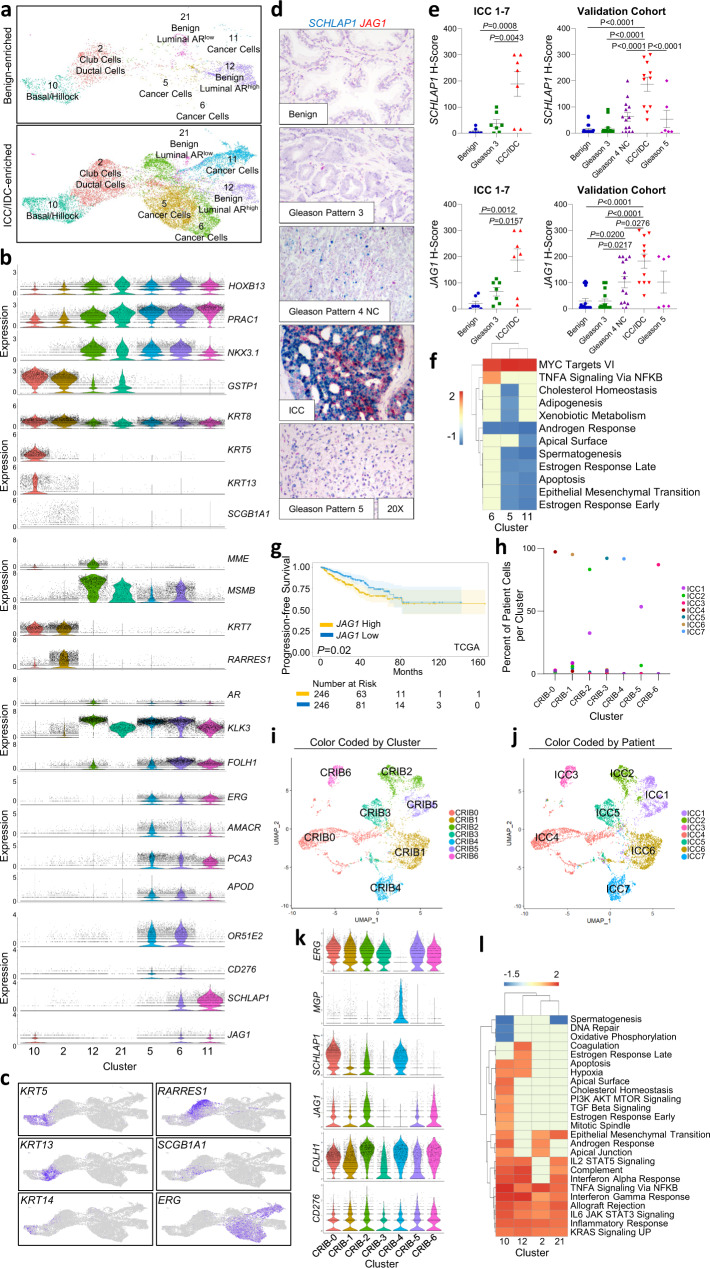
Increased *SCHLAP1* and *JAG1* in prostate ICC/IDC. **a** Unsupervised graph-based clustering of epithelial cell clusters (2, 5, 6, 10, 11, 12, 21) separated by benign-enriched and ICC/IDC-enriched prostate. **b**, **c** Violin (**b**) and feature (**c**) plots of gene expression in epithelial cell clusters. **d**, **e** Representative images at 20x, bar = 10 µm (**d**) and quantification (**e**) of *SCHLAP1* and *JAG1* expression by RNAscope in ICC1-7 at RP for benign prostate luminal epithelial cells, Gleason pattern 3 prostate cancer, and ICC (*n* = 7 biologically independent samples). Quantification of *SCHLAP1* and *JAG1* in an extended validation RP cohort of benign prostate luminal epithelial cells (*n* = 20) as well as Gleason pattern 3 (*n* = 18), Gleason pattern 4 non-ICC (NC) (*n* = 14), ICC/IDC (*n* = 11), and Gleason pattern 5 (*n* = 6) prostate cancer. A total of *n* = 23 biologically independent samples were assessed for *SCHLAP1* and *JAG1* expression with samples having more than one histology. Quantification of *SCHLAP1* and *JAG1* expression by H-score (intensity x percent expression). Graphs are shown as mean ± SEM and analyzed by one-way ANOVA with Tukey’s multiple comparisons. **f** PssGSEA of hallmark pathways altered in ICC/IDC cancer cells (clusters 5, 6, and 11) compared to benign luminal epithelial cells in cluster 12. **g** Kaplan–Meier and log-rank test of progression-free survival in the TCGA PanCancer Atlas prostate adenocarcinoma for *JAG1* by median expression (*n* = 492). **h** Percent of patient cells per cluster after re-clustering clusters 5, 6, and 11 into 7 clusters (CRIB-0 through CRIB-6). **i**, **j** UMAP visualization of re-clustering of clusters 5, 6, and 11 (CRIB-0 through CRIB-6) color-coded by cluster (**i**) and by patient (**j**). **k** Violin plots of gene expression in CRIB-0 through CRIB-6. **l** PssGSEA of hallmark pathways altered in benign epithelial clusters in ICC/IDC-enriched prostate compared to benign-enriched prostate. Source data are provided as a Source Data file.

The heterogeneity of ICC/IDC cells has not been well established. Unsupervised graph-based clustering of all cells generated 7 epithelial clusters: 2, 5, 6, 10, 11, 12, and 21, as well as a small ciliated epithelial cluster (cluster 25) (Figs. 1j and 3a). Clusters 12 and 21 were significantly decreased in ICC/IDC-enriched tumors compared to benign-enriched prostate (Fig. 2c). While benign-enriched prostate cells from all patients contributed to clusters 12 and 21, fewer cells were from ICC/IDC-enriched tumors (Fig. 2c and Supplementary Fig. 3a, b). Clusters 12 and 21 were positive for acinar luminal epithelial markers (*MSMB*) but were negative for cancer cell markers (*ERG* and *AMACR*) (Fig. 3b and Supplementary Fig. 4a, genes for clusters 12 and 21 in Source Data)^36^. While cluster 12 was positive for *AR* and AR-induced genes (*KLK3*), cluster 21 had diminished expression of these genes (Fig. 3b). These findings support that both the AR high, and AR low populations of benign luminal epithelial cells were decreased in the ICC/IDC TME.

In contrast to benign luminal epithelial cells, clusters 5, 6, and 11 were principally composed of cells from ICC/IDC-enriched prostate with very minimal contribution from benign-enriched prostate. Clusters 5, 6, and 11 expressed luminal epithelial markers, *AR*, AR-induced genes, and cancer cell markers (*ERG* and *AMACR),* supporting their identity as cancer cells (Fig. 3b, c). ICC/IDC-enriched regions sampled for scRNAseq contained varying levels of ICC, IDC, Gleason pattern 4 non-ICC, and Gleason pattern 3 (Supplementary Fig. 4b, c). Collectively, data suggest clusters 6 and 11 were enriched for ICC/IDC cells while cluster 5 was enriched for Gleason pattern 3 cells and potentially a subset of Gleason pattern 4 non-cribriform cells. Cluster 11 was spatially distinct from clusters 5 and 6, and nearly all cells in cluster 11 were from one patient (ICC4) whose sampled region for scRNAseq was predominantly ICC with minimal to no adjacent IDC, Gleason pattern 4 non-ICC, or Gleason pattern 3, thereby supporting ICC as the principal cellular identity of cluster 11. Conversely, cluster 5 predominantly consisted of cells from 5 patients (ICC1, ICC2, ICC3, ICC5, and ICC6), all of whom had adjacent Gleason pattern 3 (>5%) in the region isolated for scRNAseq, whereas the other 2 patients (ICC4 and ICC7) had minimal contribution to cluster 5 and had minimal Gleason pattern 3 (<5%) detected in their isolated tissue (Supplementary Fig. 4b–d). Cells in cluster 5 were *ERG* positive, which is consistent with isolated tissue for scRNAseq containing adjacent Gleason pattern 3 (ICC1, ICC2, ICC3, ICC5, and ICC6) but not with ICC/IDC which was ERG negative in ICC/IDC from 2 of the 7 patients (Figs. 1e and 3c). Cluster 6 was composed of cells from all patients and consistent with ICC/IDC, had *ERG*^+^ and *ERG*^-^ subpopulations (Fig. 3c). Compared to cluster 5, clusters 6 and 11 had increased expression of *FOLH1*, which has been shown to be overexpressed in ICC (Fig. 3b)^37^. Similarly, *SCHLAP1*, a lncRNA associated with ICC/IDC and adverse outcomes^20,31,38,39^, was increased in clusters 6 and 11 compared to cluster 5 and benign epithelial cells (Fig. 3b). RNAscope of RP tissue from ICC1-7 and an additional extended independent cohort showed increased *SCHLAP1* expression in ICC/IDC compared to benign prostate epithelial cells and Gleason pattern 3, Gleason pattern 4 non-ICC (NC) and Gleason pattern 5 prostate cancer (Fig. 3d, e). Collectively, pathology and gene expression data support that clusters 6 and 11 were enriched for ICC/IDC while cluster 5 was likely enriched for Gleason pattern 3 prostate cancer.

Compared to benign luminal epithelial cells, cancer cells in all clusters (5, 6, and 11) were enriched for potential therapeutic targets and/or biomarkers, including *FOLH1* (PSMA)^40–42^ and *PCA3*^43,44^ (Fig. 3b). *APOD*, an oxidative stress response gene increased in ETS^+^ prostate cancers^45^, and *CD276* (B7-H3), an immune checkpoint associated with adverse prostate cancer outcomes^46^, were also elevated in cancer cell clusters (Fig. 3b). Single-sample gene-set enrichment analysis with paired comparisons (pssGSEA) was used to test for enrichment of hallmark pathways. PssGSEA indicated that the MYC Targets VI hallmark was increased in clusters 5, 6, and 11, suggesting that this alteration may be common among several Gleason patterns of prostate cancer (Fig. 3f). In support, elevated MYC expression was detected in both ICC/IDC and Gleason pattern 3 prostate cancer by IHC (Supplementary Fig. 2b). In contrast, the TNFα signaling via NFκB hallmark was increased in ICC/IDC-enriched cells in cluster 6 compared to benign luminal epithelial cells. Of the top 5 ranked TNFα signaling via NFκB hallmark genes, *JAG1*, a Notch ligand correlated with prostate cancer metastasis, angiogenesis, and reactive stroma formation^47–49^, was distinctly increased in clusters 6 and 11 compared to clusters 5 and 12 (Fig. 3b and Supplementary Fig. 4e). RNAscope of RP tissue from ICC1-7 and from an extended independent validation cohort confirmed higher *JAG1* expression in ICC/IDC compared to benign luminal epithelial cells, Gleason pattern 3, and Gleason pattern 4 non-ICC (NC) prostate cancer (Fig. 3d, e). Increased *JAG1* showed a significant, but modest association with worse prostate cancer progression-free survival in the TCGA PanCancer Atlas prostate adenocarcinoma cohort (Fig. 3g).

To define further the heterogeneity of prostate ICC/IDC cells, clusters 5, 6, and 11 were re-clustered, yielding 7 distinct clusters: CRIB-0 through CRIB-6 (Fig. 3h–k, Supplementary Fig. 4f, genes for CRIB0-6 in Source Data). Cells predominantly clustered by patient except for cells from ICC1 which were split between two clusters (Fig. 3h–j). Patient-based clustering did not occur in benign epithelial clusters (clusters 2, 10, 12) when re-clustered individually using similar parameters (Supplementary Fig. 4g). *CD276* was expressed in all clusters while *SCHLAP1* and *JAG1* expression was heterogenous between clusters with high expression in most patients (Fig. 3k). Collectively, these findings support that ICC/IDC cancer cells have high inter-patient heterogeneity, but commonly upregulate *SCHLAP1* and TNFα signaling via NFĸB pathway member *JAG1*.

### Increased inflammatory pathways in benign epithelial cells in the ICC/IDC TME

How the development of ICC/IDC impacts adjacent benign epithelial cells in the TME is not fully known. In addition to luminal epithelial cells, adult human prostate consists of several other cell types, including basal cells, rare neuroendocrine (NE) cells, and the recently described club and hillock cells^50^. Clusters 10 and 2 consisted of cells from all patients, and the relative abundance of clusters 10 and 2 were not significantly altered in ICC/IDC-enriched tumors compared to benign-enriched prostate (Fig. 2c and Supplementary Fig. 3a). Overall, both Clusters 10 and 2 had low expression of prostate cancer-associated genes*, AR*, and AR-induced genes (Fig. 3b and Supplementary Fig. 4a). Cluster 10 was enriched for *KRT5*^+^*TP63*^+^ cells with distinct subclusters of *KRT5*^+^ cells enriched for either *KRT14*^+^ basal cells or *KRT13*^+^ hillock cells (Fig. 3b, c and Supplementary Fig. 4a)^50^. Similar cell type heterogeneity was detected in cluster 2. A subcluster of cells in cluster 2 was enriched for *KRT7* and *RARRES1*, two markers of ductal luminal epithelial cells^36^, while a distinct small subcluster of cells expressed club cell markers, *SCGB1A1* and *SCGB3A1* (Fig. 3b, c, Supplementary Fig. 4a, genes for cluster 2 in Source Data). Consistent with young adult benign prostate^50^, *KRT13*^+^ (hillock) and *SCGB1A1*^+^ (club) cells were infrequently clustered in benign epithelial glands and were only rarely interspersed among cancer cells (Supplementary Fig. 5). Interestingly, cluster 2 also contained rare cells that strongly expressed NE markers (*CHGA, SCG2*, *ASCL1*, and *GRP*)^50^, thereby, supporting cluster 2 identity as a heterogenous cluster of largely ductal luminal epithelial cells and club cells but also rare NE cells (Fig. 3c)^50^.

PssGSEA showed increased androgen response in cluster 2 (club/ductal luminal) and cluster 10 (basal/hillock) cells from ICC/IDC-enriched regions compared to benign-enriched regions (Fig. 3l), which is consistent with a recent report showing increased androgen response in prostate cancer-associated club and basal cells compared to the normal club and basal cells, respectively^51^. Thus, increased androgen response in prostate cancer-associated club and basal cells may be common across multiple prostate cancer subtypes. Interestingly, club/basal/hillock cells in ICC/IDC-enriched regions had increased inflammatory hallmarks, including TNFα signaling via NFκB, IFNγ response, and IL6/JAK/STAT3 signaling compared to these cell types in benign-enriched prostate (Fig. 3l). Inflammatory hallmarks were also increased in benign luminal epithelial cells (clusters 12 and 21) from ICC/IDC-enriched regions compared to benign-enriched prostate (Fig. 3l). Collectively, these findings support that benign epithelial cells in the ICC/IDC TME had differential gene expression reflecting increased inflammatory response and signaling compared to these cell types in the benign prostate environment.

### Increased JAG1/NOTCH signaling and angiogenesis in prostate ICC/IDC

**Fig. 4:**
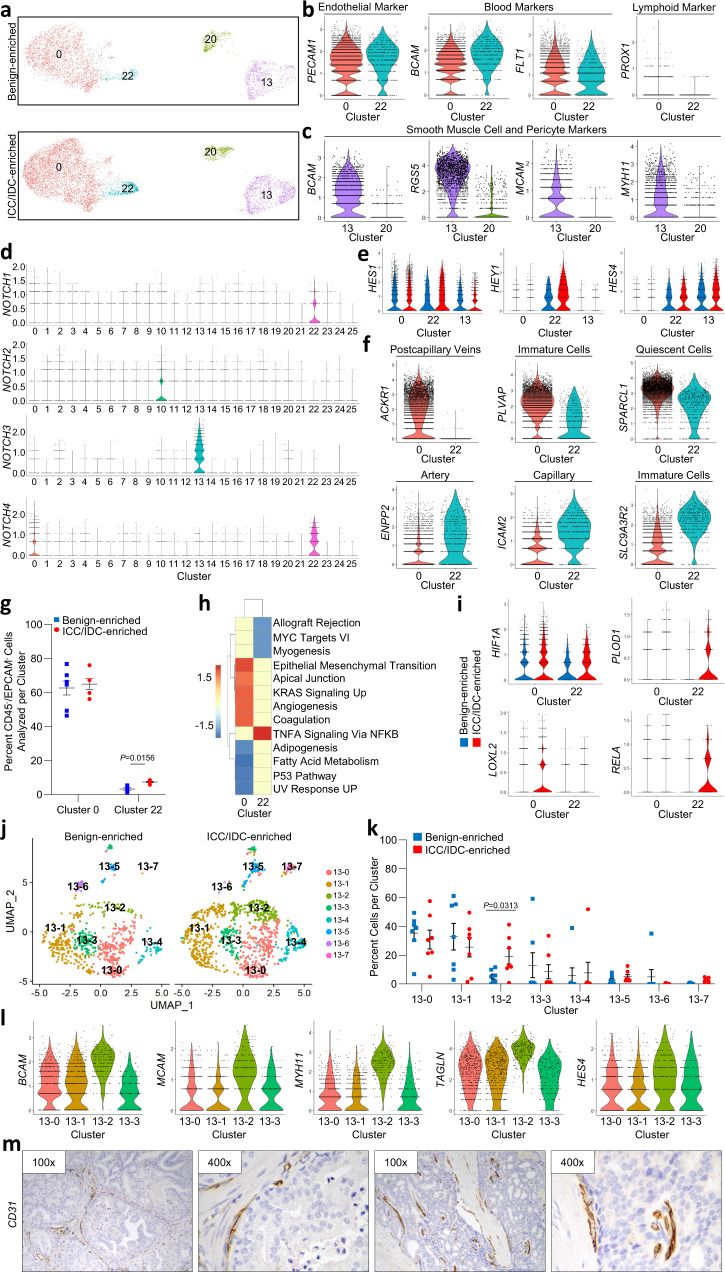
Increased JAG1/NOTCH signaling and angiogenesis in the prostate ICC/IDC TME. **a** Unsupervised graph-based clustering of all samples visualized by UMAP highlighted for endothelial clusters 0 and 22 and SMC cluster 13 delineated by benign-enriched and ICC/IDC-enriched prostate. **b** Violin plots of endothelial, blood, and lymphoid marker expression in clusters 0 and 22. **c** Violin plots of SMC and pericyte markers in clusters 13 and 20. **d** Violin plots of NOTCH receptor expression in clusters 0–25. **e** Violin plots of NOTCH-induced genes in endothelial clusters 0 and 22 and SMC cluster 13 delineated by benign-enriched and ICC/IDC-enriched prostate. **f** Violin plots of markers differentially enriched in cluster 0 compared to cluster 22**. g** Percent total of EpCAM^-^/CD45^-^ benign-enriched and ICC/IDC-enriched prostate cells per endothelial cluster (0 and 22). The graph shown as mean ± SEM and analyzed by Wilcoxon matched-pair signed rank two-tailed test; *n* = 7 biologically independent samples. The graph is also shown as part of Fig. 2d. **h** PssGSEA of hallmark pathways in ICC/IDC-enriched prostate compared to benign-enriched prostate in clusters 0 and 22. **i** Violin plots of markers in ICC/IDC-enriched prostate compared to benign-enriched prostate in clusters 0 and 22. **j** UMAP visualization of cluster 13 after re-clustering. **k** Percent of cells in clusters 13–0 through 13–7. The graph is shown as mean ± SEM and analyzed by Wilcoxon matched-pair signed rank two-tailed test; *n* = 7 biologically independent samples. **l** Violin plots of markers in clusters 13–0 through 13–3. **m** Representative images of CD31 expression by IHC in ICC/IDC prostate cancer from RP (*n* = 7 biologically independent samples) at 100x (bar = 100 µm) and 400x. Source data are provided as a Source Data file.

How ICC/IDC impacts non-epithelial cells in the TME has not been well established. JAG1 is a cell surface ligand that activates NOTCH receptors through cell-to-cell contact with adjacent cells. Due to elevated *JAG1* in ICC/IDC cancer cells, NOTCH signaling may be increased in cells directly adjacent to ICC/IDC. Expression analyses revealed that NOTCH receptors were distinctly enriched in *PECAM1*^+^ endothelial cells (clusters 0 and 22) and in *BCAM*^+^ vascular SMC (cluster 13) (Fig. 4a–d, Supplementary Fig. 6a, b) in both the ICC/IDC TME and the benign prostate microenvironment. Specifically, *NOTCH4* was highly expressed by endothelial cells in clusters 0 and 22, *NOTCH1* was also expressed by endothelial cells in cluster 22, and *NOTCH3* was expressed by vascular SMC (cluster 13) (Fig. 4d). *NOTCH2* was enriched in cluster 10 (hillock/basal) cells from benign-enriched prostate. Consistent with elevated *JAG1* in ICC/IDC cancer cells, NOTCH target genes were significantly increased in endothelial cells in clusters 0 (*HES1*) and 22 (*HES1* and *HEY1*) and SMC (*HES4*) located the ICC/IDC TME compared to benign prostate (Fig. 4e).

Marker gene expression analysis was used to delineate endothelial identity in clusters 0 and 22. Both endothelial clusters were positive for blood markers (*BCAM*) and had a minimal expression of lymphatic markers (*PROX1),* supporting that they predominantly consisted of blood endothelial cells (Fig. 4b and Supplementary Fig. 6a). Cells within cluster 0 were characterized by the expression of endothelial markers found in postcapillary veins (*ACKR1, VWF*), immature cells (*PLVAP, IGFBP4*), and quiescent cells (*SPARCL1*), while cells within cluster 22 were characterized by the expression of markers found in arteries (*CXCL12*, *ENPP2*), capillaries (*ICAM2, IFI27, TIMP3*), and immature (*A2M*, *SLC9A3R2*, *CRIP2*) endothelial cells (Fig. 4f and Supplementary Fig. 6c, d, and genes for clusters 0 and 22 in Source Data)^52^. The abundance of endothelial cells in cluster 22, but not cluster 0, was significantly increased in ICC/IDC-enriched regions compared to benign-enriched regions (Fig. 4g). PssGSEA showed increased angiogenesis and markers of hypoxia in endothelial cells from ICC/IDC-enriched regions compared to benign-enriched prostate (Fig. 4h, i).

Consistent with increased endothelial cells and NOTCH signaling, scRNAseq analyses support that ICC/IDC-enriched regions also had increased vascular SMC. Cluster 13 expressed vascular SMC (*BCAM*) and pericyte (*RGS5*) markers (Fig. 4c). To assess for differences in these cell types between ICC/IDC-enriched and benign-enriched prostate, cluster 13 was re-clustered into 8 clusters (clusters 13–0 through 13–7) (Fig. 4j, genes for 13-0 to 13-7 in Source Data). Clusters 13–4 through 13–7, however, were small or were contributed to by only a minority of patients. Cluster 13-0 was significantly enriched for pericyte markers, while clusters 13–1 and 13–3 were significantly enriched for the expression of several transcription factors (*JUN* and *ATF3)* (Supplementary Fig. 6e). While the relative abundance of clusters 13–0, 13–1, and 13–3 were similar, cluster 13-2 was significantly increased in ICC/IDC-enriched regions compared to benign-enriched regions (Fig. 4k). Cluster 13–2 was enriched for multiple vascular SMC genes as well as the NOTCH target gene *HES4* (Fig. 4l).

IHC for CD31 (*PECAM1)* on RP sections indicated that ICC/IDC foci were associated with adjacent external vessels (Fig. 4m). However, some ICC/IDC foci had limited tumor endothelial cell (TEC) infiltration, but consistent with histologic features diagnostic of cribriform, the majority of intraglandular cells were not in contact with stroma^53^. Collectively, these findings support a model in which increased JAG1 expression in ICC/IDC cancer cells induced angiogenesis through NOTCH signaling in vascular endothelial and SMC cells.

### CAFÉ CAF are enriched in ICC/IDC and are associated with worse outcomes

**Fig. 5:**
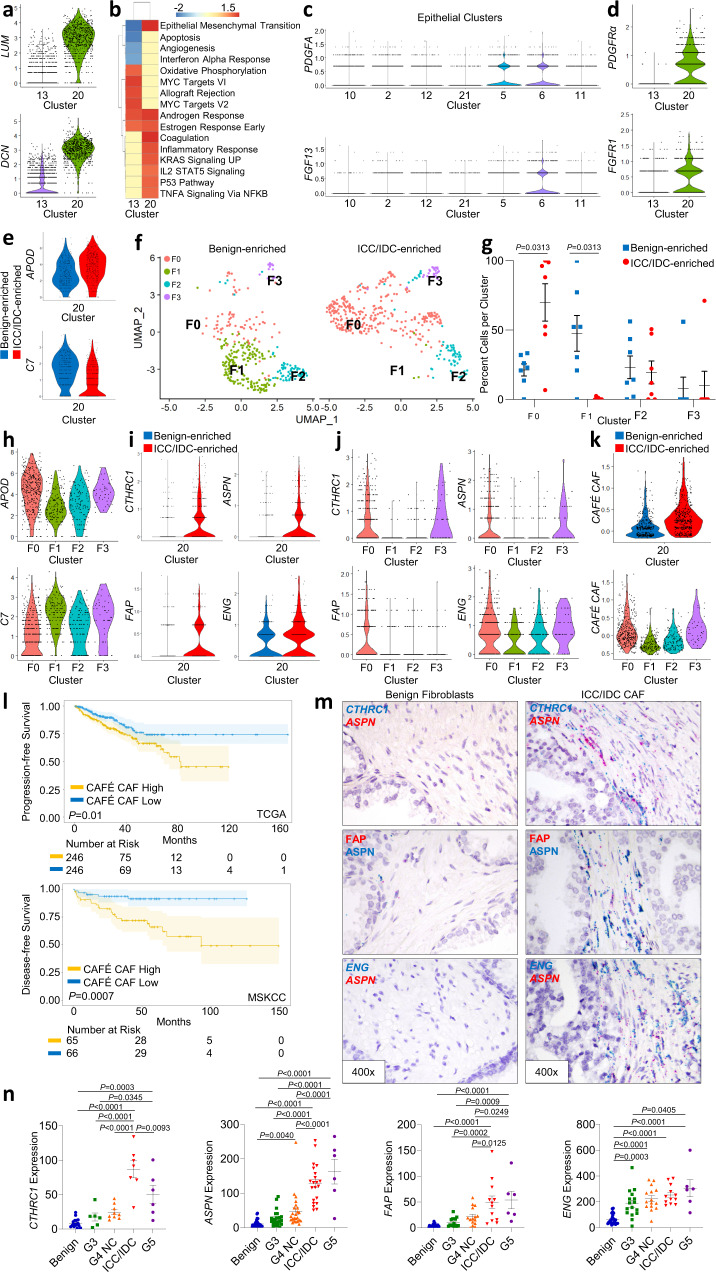
CAFÉ CAF are enriched in ICC/IDC and are associated with worse outcomes. **a** Violin plots of fibroblast marker expression in clusters 13 and 20. **b** PssGSEA of hallmark pathways altered in ICC/IDC-enriched compared to benign-enriched prostate in clusters 13 and 20. **c** Violin plots of ligand expression in epithelial clusters. **d** Violin plots of receptor expression in clusters 13 and 20. **e** Violin plots of peri-epithelial (*APOD*) and interstitial (*C7*) fibroblast markers in ICC/IDC-enriched compared to benign-enriched prostate in cluster 20. **f** UMAP visualization of unsupervised graph-based re-clustering of cluster 20 (F0–F3) separated by benign-enriched and ICC/IDC-enriched prostate. **g** Cell percentage per cluster from ICC/IDC-enriched and benign-enriched regions after re-clustering cluster 20 (F0–F3). The graph is shown as mean ± SEM and analyzed by Wilcoxon matched-pair signed rank two-tailed test; *n* = 7 biologically independent samples. **h** Violin plots of marker gene expression in clusters F0-F3. **i**, **j**, **k** Violin plots of CAFÉ CAF gene expression in cluster 20 differentiated by ICC/IDC-enriched and benign-enriched prostate (**i**, **k**) and in clusters F0–F3 (**j**, **k**). **l** Kaplan–Meier and log-rank test of progression-free survival in the TCGA PanCancer Atlas Prostate Adenocarcinoma (*n* = 492) for the CAFÉ CAF signature. Kaplan–Meier and log-rank test of disease-free survival in the MSKCC Prostate Adenocarcinoma for the CAFÉ CAF signature (*n* = 131). **m**, **n** Representative images at 400x, bar = 10 µm (**m**) and quantification (**n**) of expression in a combined RP prostate cancer cohort of new and historical^33^ samples adjacent to benign prostate (*CTHRC1* *n* = 17, *ASPN* *n* = 40, *FAP* *n* = 27, and *ENG* *n* = 26), Gleason pattern 3 (*CTHRC1* n = 6, *ASPN* *n* = 24, *FAP* *n* = 15, and *ENG* *n* = 16), Gleason pattern 4 non-ICC (G4 NC; *CTHRC1* *n* = 9, *ASPN* *n* = 24, *FAP n* = 15, and *ENG* *n* = 15), ICC/IDCC (*CTHRC1* *n* = 7, *ASPN* *n* = 21, *FAP* *n* = 12, and *ENG* *n* = 12), and Gleason pattern 5 (G5; *CTHRC1* *n* = 6, *ASPN* *n* = 6, *FAP* *n* = 6, and *ENG* *n* = 6) prostate cancer. *N* = 42 biologically independent samples were used to assess *CTHRC1*, *ASPN*, *FAP*, and *ENG* expression with samples having more than one histology for assessment and overlap between markers. Graphs are shown as mean ± SEM and analyzed by one-way Anova with Tukey’s Multiple Comparisons. Source data are provided as a Source Data file.

JAG1-NOTCH2 signaling between breast cancer cells and fibroblasts was shown to impact CAF phenotypes^54^, however, minimal expression of NOTCH and NOTCH-induced genes by CAF in the ICC/IDC TME indicates that alternative mechanisms drive their activation (Fig. 5a, b). Instead, ligand/receptor analyses suggest that increased *PDGFA* and *FGF13* expression by ICC/IDC cancer cells (cluster 6) may impact CAF phenotypes through *PDGFRα* and *FGFR1* in fibroblasts (cluster 20)^55^ (Fig. 5c, d).

Multiple studies have begun to elucidate fibroblast heterogeneity in several cancer types, including breast and pancreatic cancer^33,56–60^. A recent study identified two fibroblast subtypes in benign human prostate: an *APOD*^+^ peri-epithelial subtype and a *C7*^+^ interstitial subtype^61^; however, CAF heterogeneity has not been fully delineated in prostate cancer^33^. ICC/IDC-enriched CAF in cluster 20 were significantly increased for *APOD* and significantly decreased for *C7* expression compared to benign-enriched fibroblasts (Fig. 5e). To determine if peri-epithelial fibroblasts were indeed the fibroblast subtype enriched in ICC/IDC regions, cluster 20 was re-clustered into four clusters (F0-F3) (Fig. 5f, g, genes for F0-F3 in Source Data). Cluster F0 was delineated by higher *APOD* expression and was significantly increased in ICC/IDC-enriched regions compared to benign-enriched prostate (Fig. 5f–h). In contrast, cluster F1 was marked by higher *C7* expression and was significantly decreased in ICC/IDC-enriched regions compared to benign-enriched prostate.

CAF from ICC/IDC-enriched regions examined both prior to and after re-clustering were significantly elevated for the expression of genes associated with adverse pathology, poor outcomes, and/or immunosuppression, including *TNC*^62^, *TGFB1*^63^, *SFRP4*^64^, *CCL2*^65^, *CTHRC1*^66,67^, *ASPN*^33,68,69^, *FAP*^34,35,58,70^, and *ENG*^71–73^ (Fig. 5i, j and Supplementary Fig. 7a). A 4-gene signature based on ICC/IDC CAF markers: *CTHRC1, ASPN, FAP, and ENG* (CAFÉ CAF), showed a significant association with worse prostate cancer progression-free survival in the TCGA PanCancer Atlas prostate adenocarcinoma cohort^74^ and worse disease-free survival in the MSKCC Prostate Adenocarcinoma cohort^75^ (Fig. 5k, l). *CTHRC1*^+^, *ASPN*^+^, *FAP*^+^, and *ENG*^+^ CAF spatial dynamics and associations with other prostate cancer grades and/or histological subtypes were examined by RNAscope in an independent extended RP prostate cohort of combined new and historical samples^33^. *CTHRC1*^*+*^, *ASPN*^*+*^, and *FAP*^*+*^ CAF were located peri-epithelial to ICC/IDC and were significantly enriched in ICC/IDC compared to benign prostate as well as Gleason pattern 3 and Gleason pattern 4 non-ICC prostate cancer (Fig. 5m, n and Supplementary Fig. 7c, d). While *CTHRC1*^*+*^ CAF were slightly elevated, *ASPN*^*+*^ and *FAP*^*+*^ CAF were comparable between ICC/IDC and Gleason pattern 5 prostate cancer. *ENG*^+^ CAF were significantly elevated in all cancer grades/histological subtypes examined compared to benign prostate. These results support that *CTHRC1*^*+*^, *ASPN*^*+*^, and *FAP*^+^ CAF were increased in ICC/IDC and Gleason pattern 5 prostate cancer, while *ENG*^*+*^ CAF were increased in cancer. Collectively, these findings support that CAF in the ICC/IDC TME express peri-epithelial fibroblasts markers, have common gene expression as CAF adjacent to Gleason pattern 5 prostate cancer, and are associated with worse outcomes.

### Immune exclusion and reduced T cell fraction and clonality in the prostate ICC/IDC TME

**Fig. 6:**
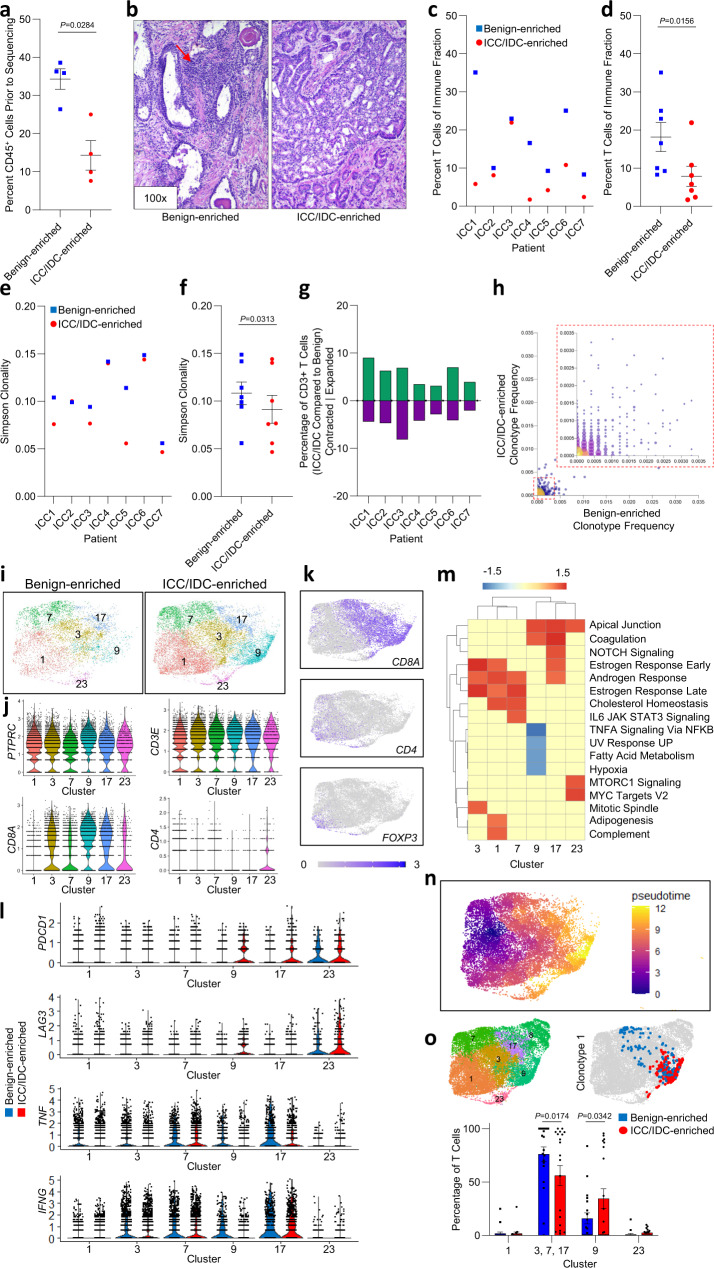
Immune exclusion, reduced T cell fraction and clonality, and increased T cell dysfunction in the prostate ICC/IDC TME. **a** Quantification of percent CD45^+^ cells by flow cytometry from paired samples of benign-enriched and ICC/IDC-enriched prostate. Graph shown as mean ± SEM analyzed by paired two-tailed *t*-test; *n* = 4 biologically independent samples. FACS gating strategies shown in Supplementary Fig. 10a. **b** Representative images of inflammatory cells (red arrow) in benign-enriched regions and IDC/ICC-enriched regions by H & E at 100x, bar = 50 µm, (*n* = 7 biologically independent samples). **c**, **d** Percent T cells of the immune fraction by individual patient (**c**) and collectively (**d**) for benign-enriched and ICC/IDC-enriched prostate. Graph in **d** shown as mean ± SEM analyzed by Wilcoxon matched-pair signed rank two-tailed test; *n* = 7 biologically independent samples. **e**, **f** Simpson Clonality by individual patient (**e**) and collective (**f**) for benign-enriched and ICC/IDC-enriched prostate. Graph in **f** shown as mean ± SEM analyzed by Wilcoxon matched-pair signed rank two-tailed test; *n* = 7 biologically independent samples. **g** Percentage of T cells contracted or expanded between benign-enriched and ICC/IDC-enriched prostate. **h** Clonotype frequency between benign-enriched and ICC/IDC-enriched prostate. **i** Unsupervised graph-based clustering of all samples visualized by UMAP highlighted for T cell clusters 1, 3, 7, 9, 17, and 23 delineated by benign-enriched and ICC/IDC-enriched prostate. **j**, **k** Violin (**j**) and feature plots (**k**) of clusters 1, 3, 7, 9, 17, and 23 for immune and T cell markers. **l** Violin plots of markers in ICC/IDC-enriched prostate compared to benign-enriched prostate in clusters 1, 3, 7, 9, 17, and 23. **m** PssGSEA of hallmark pathways in ICC/IDC-enriched prostate compared to benign-enriched prostate in T cell clusters. **n** Pseudotime trajectory analysis for clusters 1, 3, 7, 9, 17, and 23. **o** Representative clonotype cluster location in benign-enriched and ICC/IDC-enriched prostate and quantification. The graph is shown as mean ± SEM analyzed by Wilcoxon matched-pair signed rank two-tailed test; *n* = 7 biologically independent samples. Source data are provided as a Source Data file.

Expansion of a CAF subtype expressing immunosuppressive markers, including *FAP*^34,35^ suggests that the ICC/IDC TME may be associated with dysfunctional T cells; however, little has been reported about the immune TME associated with prostate ICC/IDC. To better determine the immune repertoire and heterogeneity in the ICC/IDC TME compared to benign regions, CD45^+^ cells were analyzed by scRNAseq and T cells were analyzed by TCR sequencing. Flow cytometry analysis prior to sequencing showed a significant decrease in CD45^+^ cells in ICC/IDC-enriched regions compared to benign-enriched regions (Fig. 6a, b). Normalization of CD45^+^, EpCAM^+,^ and CD45^-^/EpCAM^-^ fractions detected by flow cytometry prior to sequencing with the number of TCR^+^ cells after sequencing, indicated that within the immune fraction, significantly fewer T cells were detected in ICC/IDC-enriched tumors compared to benign-enriched prostate (Fig. 6c, d). In addition to fraction, T cells in the ICC/IDC TME were examined for diversity by analyzing clonotype richness (percent of different clonotypes) and evenness (percent distribution of each clonotype) by Simpson clonality. Simpson clonality was significantly decreased in ICC/IDC-enriched compared to benign-enriched prostate, thereby indicating a more even distribution of clonotypes in ICC/IDC-enriched regions (Fig. 6e, f). Richness (percent of different clonotypes), however, was similar between ICC/IDC-enriched and benign-enriched prostate (T cell richness in Source Data). Differences in TCR clonotype repertoire were detected and approximately 5-15% of clonotypes had a two-fold or greater expansion/contraction in ICC/IDC-enriched compared to benign-enriched regions (Fig. 6g, h). These data support that ICC/IDC-enriched prostate had diminished immune infiltration, and of the immune cells, the T cell fraction was reduced and had decreased clonality.

### Increased dysfunctional markers in CD8^+^ T cells in the prostate ICC/IDC TME

Recent single-cell analyses have provided insight into the substantial heterogeneity in intratumoral T cell states that likely exist along a continuum^76–78^. Due to the lack of a consensus nomenclature for human T cell states analyzed by scRNAseq, Van der Leun et al. integrated multiple scRNAseq studies to broadly categorize CD8^+^ T cell states as naïve-like, predysfunctional (effector memory, memory, and transitional), cytotoxic (effector), and dysfunctional (exhausted)^77^. Unsupervised graph-based clustering of all cells generated six *CD45*^*+*^*CD3*^+^ clusters with contributions from all patients (Fig. 6i, k and Supplementary Fig. 3a). Mapping showed a heterogenous cluster (cluster 1) that expressed naïve-like markers including *CD3*, *IL7R*, *CCR7*, and *SELL*, which were similar between ICC/IDC-enriched and benign-enriched T cells (Fig. 6j and Supplementary Fig. 8a, genes for cluster 1 in Source Data)^76–79^.

*CD8* + T cells mapped to 5 of the 6 T cell clusters: 3, 7, 9, 17, and 23 (Fig. 6j, k). Both ICC/IDC-enriched and benign-enriched *CD8*^*+*^ T cells in clusters 3, 7, and 17 expressed the predysfunctional marker *GZMK* (Supplementary Fig. 8a). Compared to clusters 3 and 17, *CD8*^+^ T cells in cluster 7 expressed lower levels of granzymes and perforin but were notable for high levels of human stress-activated protein (Supplementary Fig. 8a). ICC/IDC-enriched *CD8*^*+*^ T cells in cluster 7 had decreased expression of *IFNG* and increased expression of *PDCD1* (Fig. 6l, DEG analysis for cluster 7 in Source Data). Cluster 3 cells expressed *PRF1, GZMA*, *GZMH, TNF*, and *IFNG*; however, expression of *IFNG* and *TNF* were lower in ICC/IDC-enriched compared to benign-enriched *CD8*^*+*^ T cells (Fig. 6l and Supplementary Fig. 8a)^77^. *CD8*^*+*^ T cells in cluster 17 were distinguished by high *CCL4* expression and additionally expressed *GZMA*, GNLY, *PRF1*, *TNF*, and *IFNG* suggesting that cluster 17 cells fell on the spectrum toward cytotoxic *CD8*^*+*^ T cells. Cluster 17 *CD8*^*+*^ T cells from ICC/IDC-enriched prostate had increased expression of dysfunctional markers *PDCD1* and *LAG3* and decreased *TNF* and *IFNG* expression (Fig. 6l, DEG analysis for cluster 17 in Source Data). Like cluster 17 cells, cluster 9 cells also expressed *GZMA, GZMB, GZMH, GNLY*, and *PRF1*. *CD8*^*+*^ T cells in ICC/IDC-enriched prostate had a higher expression of *PDCD1* and *LAG3* and decreased *IFNG*, thereby suggesting that cluster 9 *CD8*^*+*^ T cells in benign-enriched regions were more cytotoxic while cluster 9 *CD8*^*+*^ T cells in ICC/IDC-enriched regions were more dysfunctional (Fig. 6l, DEG analysis for cluster 9 in Source Data). PssGSEA indicated that ICC/IDC-enriched T cells in cluster 9 had decreased TNFα signaling via NFκB, thereby supporting their reduced effector activity in the TME (Fig. 6m). Cells in cluster 23 also expressed markers of dysfunctional *CD8*^*+*^ T cells (*PDCD1* and *LAG3*) that were slightly higher in ICC/IDC-enriched T cells. Consistent with dysfunctional cells, cluster 23 cells expressed lower levels of granzymes, *TNF*, and *IFNG* (Fig. 6l and Supplementary Fig. 8a). Pseudotime trajectory analysis showed progression of cells from naïve cells in cluster 1 to predysfunctional cells in clusters 7/3/17, to cytotoxic cells in cluster 9 and lastly to dysfunctional cells in cluster 23 (Fig. 6n). Mapping of the top 20 TCR clonotypes showed a significant shift in several *CD8*^*+*^ T clonotypes found in benign-enriched clusters 3, 7, and/or 17 to cluster 9 in ICC/IDC-enriched prostate (Fig. 6o and Supplementary Fig. 9a–c). Overall, these findings support that CD8^+^ T cells in the ICC/IDC TME expressed decreased effector cytokines and increased dysfunctional markers.

In contrast to *CD8*^*+*^ T cells, *CD4*^*+*^ T cells mapped to three of the six T cell clusters. *CD4*^+^ T cells mapped to clusters 1, 7, and 23 (Fig. 6j, k). Treg markers (*CD4, FOXP3, IL2RA, CTLA4*) mapped to a distinct subset of these cells in cluster 1 as well as in cluster 23 (Fig. 6k and Supplementary Fig. 8b). These findings support heterogeneity in *CD4*^*+*^ Treg cells in the prostate with some having features closer to naïve T cells while others having features closer to dysfunctional T cells. Expression of *TIGIT* was increased in cluster 1 T cells in ICC/IDC compared to benign-enriched prostate (Supplementary Fig. 8a, b). Collectively, these findings indicate that ICC/IDC-enriched tumors had fewer infiltrating immune cells, and of the immune cells, the T cell fraction was lower, had less clonality, and had higher expression of exhausted markers compared to benign-enriched prostate.

### Increased *C1QB*^+^*TREM2*^+^*APOE*^+^ M2 macrophages in prostate ICC/IDC TME

**Fig. 7:**
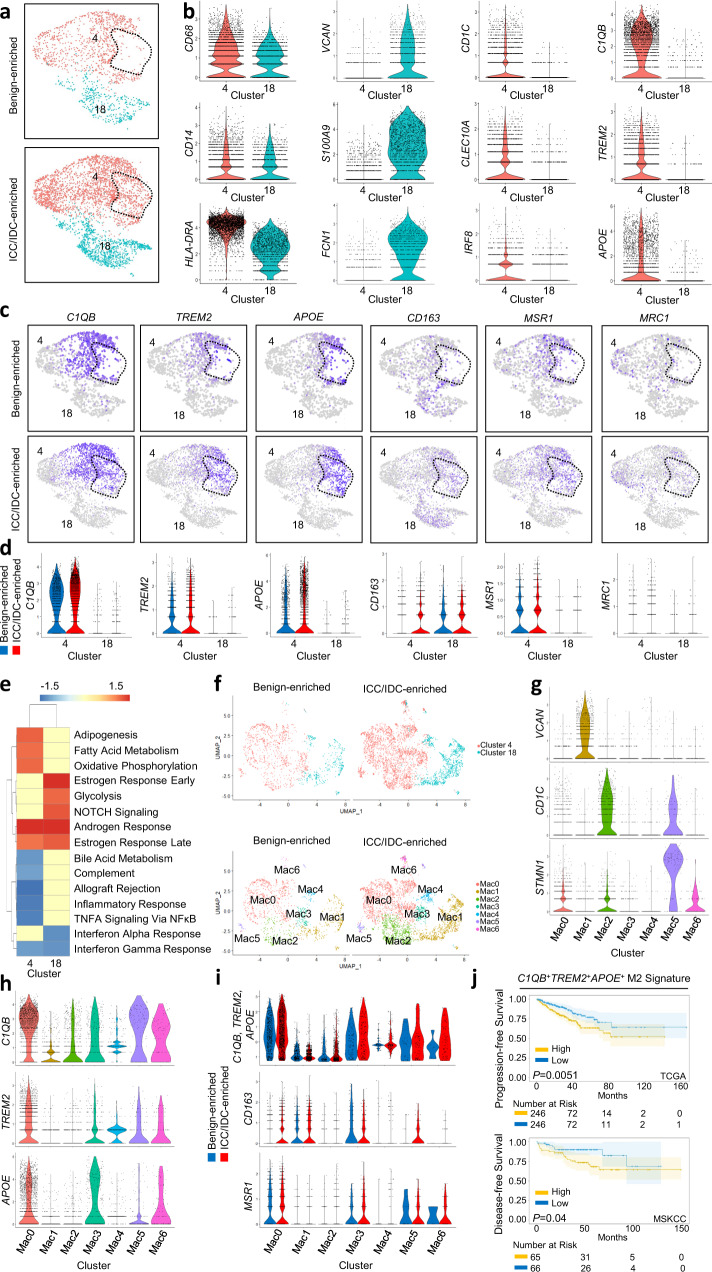
Increased *C1QB*^+^*TREM2*^+^*APOE*^+^ M2 macrophages in prostate ICC/IDC. **a** Unsupervised graph-based clustering of all samples visualized by UMAP highlighted for myeloid clusters 4 and 18 delineated by benign-enriched and ICC/IDC-enriched prostate. The dotted area demarks a subcluster of cells increased in ICC/IDC-enriched regions compared to benign-enriched regions. **b** Violin plots of myeloid, monocyte, dendritic cell, and macrophage marker expression in clusters 4 and 18. **c** Feature plots of *C1QB, TREM2, APOE,* and M2 macrophage markers (*CD163, MSR1*, and *MRC1*) in clusters 4 and 18. **d** Violin plots of *C1QB, TREM2, APOE,* and M2 macrophage markers (*CD163, MSR1*, and *MRC1*) in clusters 4 and 18 separated by ICC/IDC-enriched and benign-enriched. **e** PssGSEA of hallmark pathways in ICC/IDC-enriched prostate compared to benign-enriched prostate cells in clusters 4 and 18. **f** Re-clustering of clusters 4 and 18 into six clusters (Mac0-Mac6) separated by ICC/IDC-enriched and benign-enriched prostate and demarked by the original clusters 4 and 18. **g**–**i** Violin plots of markers in Mac0-Mac6. **j** Kaplan–Meier and log-rank test of progression-free survival in the TCGA PanCancer Atlas Prostate Adenocarcinoma for *C1QB*, *TREM2*, *APOE*, *CD163*, *MRC1*, and *MSR1* signature (*C1QB*^*+*^*TREM2*^*+*^*APOE*^*+*^ M2 Signature) by median expression (*n* = 492). Kaplan-Meier and long-rank test of disease-free survival (DFS) in the MSKCC Prostate Adenocarcinoma for *C1QB*, *TREM2*, *APOE*, *CD163*, *MRC1*, and *MSR1* signature (*C1QB*^*+*^*TREM2*^*+*^*APOE*^*+*^ M2 Signature) by median expression (*n* = 131).

While our data indicate that T cells were largely excluded or suppressed, it is not known if myeloid cells also contribute to a pro-tumorigenic immune microenvironment in ICC/IDC. CTHRC1, which is highly expressed by CAF in the ICC/IDC TME, has been shown to polarize macrophages to the M2, pro-tumorigenic, phenotype through TGF-β signaling^67^. To determine if ICC/IDC was associated with increased M2 macrophages, clusters were analyzed for myeloid lineage markers, including *CD68* (Fig. 7a, b). While monocyte markers (*VCAN* and *S100A9*) mapped to cluster 18, cluster 4 cells expressed dendritic cell markers (*CD1C* and *CLEC10A*) as well as macrophage markers associated with disease recurrence in clear cell renal cell carcinoma (*C1QB*, *TREM2*, and *APOE*)^80^ (Fig. 7b). A subcluster of cells within cluster 4 was notably increased in ICC/IDC-enriched prostate cancer compared to benign-enriched prostate. These cells were *C1QB*^+^, *TREM2*^+^, and *APOE*^+^ and expressed the anti-inflammatory M2 macrophage markers *CD163*, *MSR1*, and *MRC1* (Fig. 7c). *C1QB*, *TREM2*, *APOE*, *CD163*, and *MSR1* were significantly increased in ICC/IDC-enriched compared to benign-enriched cluster 4 cells (Fig. 7d and DEG analysis for cluster 34 in Source Data). Consistent with a M2 anti-inflammatory phenotype, cells in cluster 4 from the ICC/IDC TME had decreased inflammatory-related hallmarks by pssGSEA (Fig. 7e). To better delineate myeloid heterogeneity in the ICC/IDC TME, clusters 4 and 18 were re-clustered to 7 clusters (Mac0-6) with Mac1 and Mac4 almost entirely derived from cluster 18 while Mac0, Mac2, Mac3, Mac5, and Mac6 were largely derived from cluster 4 (Fig. 7f, genes for Mac0-Mac6 in Source Data). Monocyte markers mapped to cluster Mac1, while dendritic cell markers mapped to cluster Mac2 with a proliferative subset (*STMN1*^+^*MKI67*^+^) in Mac5 (Fig. 7f, g). *C1QB*^+^*TREM2*^+^*APOE*^+^ macrophages mapped to Mac0, which were increased along with *CD163* and *MSR1* in the ICC/IDC TME (Fig. 7h, i). A gene signature based on these cells (*C1QB, TREM2, APOE, CD163, MRC1*, and *MSR1*) showed a significant association with worse prostate cancer progression-free survival in the TCGA PanCancer Atlas prostate adenocarcinoma cohort^74^ and worse disease-free survival in the MSKCC Prostate Adenocarcinoma cohort^75^ (Fig. 7j). Overall, *C1QB*^+^*TREM2*^+^*APOE*^+^ macrophages that express M2 macrophage markers, *CD163* and *MSR1*, were increased in the ICC/IDC TME.

## Discussion

This investigation revealed an interplay between cancer cell intrinsic and microenvironmental factors that, together, contribute to the aggressive nature of ICC/IDC. Several cancer cell intrinsic pathways were commonly altered in ICC/IDC. Consistent with prior reports^16,19^, MYC-induced genes were upregulated in ICC/IDC and Gleason pattern 3 cancer cells, supporting a strong role for this pathway across multiple Gleason patterns of prostate cancer. Our findings additionally show strong enrichment in TNFα signaling via NFĸB in ICC/IDC. TNF has been shown to have pleiotropic functions in the prostate largely depending on cytokine concentration and cellular context^81,82^. Nonetheless, a NFĸB-based signature has been shown to be predicative of worse prostate cancer outcomes^83^, and studies support a role for NFĸB in prostate cancer stem-like cells^84^. In support of this, *JAG1*, a member of the TNFα signaling via NFĸB hallmark, was enriched in ICC/IDC compared to lower Gleason patterns, other Gleason pattern 4 histological subtypes of prostate cancer, and benign prostate luminal epithelial cells. Collectively, this study suggests that this pathway may contribute to ICC/IDC aggressiveness.

In addition to altered pathways, several molecules were strongly upregulated in ICC/IDC, and have implications for therapeutic opportunities. *SCHLAP1* was highly expressed in several patients and distinctly associated with ICC/IDC compared to other Gleason patterns and histological subtypes of Gleason pattern 4. Prior reports have shown that *SCHLAP1*, a long non-coding RNA associated with progression to metastasis^29–31^, is enriched in cribriform morphology^20,29^ and PTEN deficient^85^ prostate cancers. Patients with *SCHLAP1* high ICC/IDC have worse outcomes than patients with *SCHLAP1* low ICC/IDC, suggesting that *SCHLAP1* contributes to ICC/IDC aggressiveness^20,29^. PSMA/*FOLH1*^40,86^ and B7-H3/*CD276*^87^, two promising therapeutic targets, were expressed on ICC/IDC cancer cells from all patients. Radionuclide therapies using a small molecule against PSMA (PSMA-617) labeled with ^177^Lu have shown clinical benefit in two randomized clinical trials, VISION^41^ and TheraP^42^, for metastatic castration-resistant prostate cancer (mCRPC) and many other PSMA-directed therapies are quickly evolving. B7-H3 is an immune checkpoint associated with poor prostate cancer outcomes^46^. Phase 1/2 clinical trials are currently evaluating an antibody-drug conjugate against B7-H3 in combination with immunotherapy in several cancers, including mCRPC (NCT03729596, NCT05293496). Our findings indicate that despite remarkable inter-patient heterogeneity, some features of ICC/IDC universally express viable therapeutic targets for which promising therapies are currently being studied in clinical trials.

Extending beyond genomic heterogeneity in ICC/IDC cancer cells, we also revealed significant cellular diversity in the ICC/IDC TME. Stromal cell types, including vasculature and vascular SMC were altered in ICC/IDC tumors. Vascular SMC and arterial endothelial cells were significantly increased in the ICC/IDC TME, likely through JAG1/NOTCH signaling between ICC/IDC cells and vascular cells. Based on prior studies, most ICC/IDC cancer cells were thought to be distant from the vasculature^32^. Our findings indicate that most foci were associated with only peri-epithelial vasculature, while some foci had limited TEC infiltration. Collectively, these findings indicate that the ICC/IDC TME is associated with increased vasculature, but the degree of neovascular intraglandular infiltration is limited.

These studies have also begun to elucidate CAF origin and heterogeneity in the prostate ICC/IDC TME. Our findings demonstrate that a heterogenous population of CAF densely circumscribed ICC/IDC foci. Due to their location and gene expression, it is likely that these CAF originated in part from *APOD*^+^ peri-epithelial fibroblasts and not from *C7*^+^ interstitial fibroblasts^61^. ICC/IDC-associated CAF were enriched for several proteins associated with poor outcomes and/or immunosuppression, including *CTHRC1*^66^, *ASPN*^33,68,69^, *FAP*^34,70,88^, and *ENG*^71–73^. Indeed, our ICC/IDC CAFÉ CAF gene signature was associated with worse prostate cancer outcomes. Prostate ICC/IDC CAFÉ CAF shared select marker genes (*ASPN* and *FAP*) with extracellular matrix-myofibroblastic CAF (ecm-myCAF), an immunosuppressive CAF subtype detected in breast cancer and shown to be enriched in melanoma and non-small cell lung cancers (NSCLC) that did not respond to anti-PD-1 immunotherapies^58^. Thus, CAFÉ CAF may function similarly in the TME as breast ecm-myCAF. Future studies will be needed to further characterize ICC/IDC CAF heterogeneity and to determine if CAFÉ CAF have a causal role in immunosuppression and/or resistance to checkpoint inhibitor immunotherapies.

Consistent with the immunosuppressive functions of CAF^55^, the studies herein indicate that antitumorigenic immune cells were suppressed while pro-tumorigenic immune cells were enriched in the ICC/IDC TME. The association of tumor-infiltrating lymphocytes (TILS) with patient outcomes is dependent on TIL number, type, and location^89,90^. ICC/IDC was associated with decreased immune infiltration and a lower fraction of T cells; thereby suggesting that factors in the ICC/IDC TME promote T cell exclusion. CAFÉ CAF were enriched for the expression of *FAP* and *TGFB1*, both of which have been shown to mediate T cell exclusion and resistance to anti-PD-L1 therapy^35,91,92^. Recent clinical trials are assessing targeting FAP or the TGF-β pathway in combination with immune checkpoint inhibitors (NCT03875079, NCT02423343, and NCT04064190). T cells in the ICC/IDC TME were examined for diversity by analyzing clonotype richness (percent of different clonotypes) and evenness (percent distribution of each clonotype) by Simpson clonality. T cell richness was not significantly altered, but Simpson clonality was significantly decreased, suggesting that monoclonal T cell expansion does not dominate in the ICC/IDC TME. How intra-tumoral T cell diversity impacts therapeutic response has not been definitively determined^89^. A Phase 2 clinical trial (NCT02259621) of neo-adjuvant anti-PD-1 (Nivolumab) in NSCLC supports that increased T cell clonality was associated with reduced percent residual tumor at surgery^93^. In addition to alterations in clonality and number, ICC/IDC TME was associated with increased markers of T cell exhaustion. T cell exhaustion has been postulated to occur as a continuum of T cell states with different levels of functionality^77,89,94,95^. Compared to benign-enriched regions, cluster 9 ICC/IDC-enriched *CD8*^+^ T cells had increased *PDCD1* and *LAG3* levels, but comparable granzyme and perforin levels suggesting that in the ICC/IDC TME, these cells reside on the spectrum closer to an exhausted state. Trajectory analyses support this continuum and indicate that *CD8*^+^ T cell activation occurs prior to exhaustion in the TME of prostate ICC/IDC.

In addition to altered T cells, the ICC/IDC TME was associated with elevated macrophage expression of *CD163 and MSR1*, markers of pro-tumor M2 macrophages. *CD163*^+^ M2 macrophages correlate with worse prostate cancer clinicopathologic characteristics and outcomes^96,97^. *CD163* and *MSR1* were elevated in a subset of *C1QB*^+^*TREM2*^+^*APOE*^*+*^ macrophages^80^ in ICC/IDC. Similar to recent findings in kidney cancer^80^, these cells were associated with worse outcomes. A Phase 1 clinical trial (NCT0461375) is assessing PY314, a monoclonal antibody to TREM2, in combination with Pembrolizumab in advanced solid tumors. Our findings herein support that multiple immune alterations in ICC/IDC TME contribute to overall immunosuppression, and the ICC/IDC TME may model why checkpoint inhibitor monotherapies have largely been ineffective in men with prostate cancer^98,99^.

In summary, this study presents a compendium of information on both cancer and TME cells in prostate ICC/IDC. Our findings support that ICC/IDC have an aggressive phenotype by upregulating the TNFα pathway via NFκB pathway leading to the expression of *JAG1* which likely induces neovasculature through NOTCH signaling. In addition, we show that the ICC/IDC TME is immunosuppressed, resulting in less and dysfunctional T cells, as well as increased M2 *C1QB*^+^*TREM2*^+^*APOE*^*+*^ macrophages. Our findings highlight the complexity of ICC/IDC and its association with multiple adverse features that likely contribute to poor outcomes; defining the underpinnings to this aggressive subtype is essential to developing precision clinical management strategies. This study sheds light on numerous potential therapeutic vulnerabilities that could impact and positively affect clinical outcomes for patients with ICC/IDC.

## Methods

### Patients

This study protocol was approved by the Vanderbilt University Medical Center (VUMC) Institutional Review Board (IRB) (Nashville, TN). Written informed consent was obtained for all patients prior to enrollment by the Cooperative Human Tissue Network at VUMC. Patients were not compensated for participation. This study adhered to the Declaration of Helsinki principles. Over 10 months, 224 patients who were scheduled for a RP at VUMC for histologically confirmed prostate adenocarcinoma were screened by electronic medical record (EMR) for the presence of cribriform morphology as either ICC and/or IDC on their biopsy. Fourteen patients were prospectively enrolled for scRNAseq studies. Seven patients were excluded due to prostate volume at RP below threshold for research sampling or inability to locate adequate ICC/IDC for scRNAseq. This study did not distinguish between small and large cribriform patterns due to the lack of consensus on diagnostic criteria^12^. Paired benign-enriched and ICC/IDC-enriched prostate was isolated from 7 patients for scRNAseq. Patients did not have prior treatment for prostate cancer. Prostate cancer was graded in accordance with the 2014 International Society of Urological Pathology (ISUP) Guidelines^100^.

### Prostate tissue acquisition

Following surgical removal, the prostate was sectioned according to standard of care from the apex to the base in 5 mm slices (for example: slice 1-8). Each slice was then divided into quadrants (right and left anterior, right and left posterior). The sections were then entirely submitted for histologic evaluation as follows:

Slice 1, right anterior

Slice 1, right posterior

Slice 2, right anterior

Slice 2, right posterior

Slice 3, right anterior

Slice 3, right posterior

…..

Slice 1, left anterior

Slice 1, left posterior

Slice 2, left anterior

Slice 2, left posterior

Slice 3, left anterior

Slice 3, left posterior

Prostate regions potentially containing tumor were identified by firm texture and pallor compared to benign prostate. Once the tumor was identified, benign prostate was harvested at least two slices away (10 mm) from the tumor of interest. For example, if the tumor was in slice 1, the benign tissue was not taken any closer than slice 3. Tissue was isolated from potential tumor and benign regions, placed in OCT medium, frozen, cut in 5-micron sections, stained by H&E, and then evaluated by a genitourinary surgical pathologist to confirm benign tissue and cribriform tumor morphology. Following confirmation of ICC/IDC-enriched and benign-enriched samples, 0.15–0.5 g of tumor-enriched and benign-enriched tissue were harvested fresh from the prostate gland and collected in phenol red-free RPMI 1640. Benign-enriched tissue was assessed for normal/BPH glands, atrophic glands, and areas of inflammation as defined by any cluster of stromal or periglandular inflammation with >10 inflammatory cells visually estimated as percent inflammation area/total tissue area analyzed.

### Prostate tissue dissociation

Tissues were dissociated mechanically and enzymatically into single-cell suspensions with the Tumor Dissociation Kit (human, Miltenyi Biotec) as per manufacturer’s protocol (with optimization). Specimens were cut into 1–2 mm pieces and then transferred into a gentleMACS C Tube containing a mix of Enzymes H, R, and A for the first round of digestion using the gentleMACS Octo Dissociator with Heaters on the m_imptumor_01 followed by the 37C_h_TDK2 programs. Following digestion, supernatant was then kept on ice and a fresh enzyme mix was added to the undigested tissue. The second round of dissociation was done using the m_imptumor_01 program followed by half of the 37C_h_TDK2 program. The dissociated cell suspension was passed through a 70 µm MACS SmartStrainer and cells were harvested by spinning twice at 300 x g for 7 minutes. Erythrocytes were removed with Red Blood Cell Lysis Solution (10x) as per manufacturer’s protocol (for tissue). Cell number and viability were evaluated with the Invitrogen Countess Automated Cell Counter.

### Fluorescence activated cell sorting (FACS)

Immediately after dissociation, cells were resuspended in FACS buffer (5% FBS in PBS) and Fc-blocked for 10 min. Benign-enriched tissue from ICC1, ICC3, and ICC6 and tumor-enriched tissue from ICC3 had cell yields below the threshold for sorting on multiple gates, thus only viable cells from these samples were sorted with BD FACSAria III (flow rate 1.0, efficiency >90%) after staining dead cells with DAPI (Invitrogen, #D3571, dilution 1:100) in the dark for 15 minutes at room temperature. For other patient samples, cells were stained in the dark for 30 minutes at room temperature with APC-PDGFRβ (BioLegend; #323608; clone 18A2; dilution 1:80), BV711-Ep-CAM (BioLegend; #324239; clone 9C4; dilution 1:2,500), PE/Cy7-CD45 (BioLegend; #304015; clone HI30; dilution 1:5,000) and washed 3 × 5 minutes with FACS buffer. DAPI (Invitrogen; #D3571; dilution 1:100) was then added to stain dead cells, and viable cells were sorted into EpCAM^+^ (epithelial), CD45^+^ (immune), and others (TME) populations (Supplementary Fig. 10a). BD FACS Diva 8.0.1 software was used to analyze FACS data.

### Single-cell RNA sequencing

Following FACS, each cell population (EpCAM^+^, CD45^+^, and others) was concentrated to 1000 cells/µl by centrifuging at 300 x *g* for 5 minutes at 4 **°**C. Specimens sorted into specific cell populations were recombined in a ratio of 5:6:9 (EpCAM^+^: CD45+: others). The 5:6:9 ratio was based upon several factors, including the potential number of separate cell types within each broad category as well as the percent histology of the samples. The TotalSeq-C0251 Hashtag 1 (0.15 ug antibody per 100 ul staining volume) and TotalSeq-C0252 Hashtag 2 (0.15 ug antibody per 100 ul staining volume) antibodies were used to barcode benign-enriched and ICC/IDC-enriched specimens respectively, and staining was performed concurrently with the FACS fluorescent antibodies. Benign-enriched and ICC/IDC-enriched specimens were then combined in a ratio of 3:7 (benign-enriched:tumor-enriched). 31.7k cells (20k targeted retrieval) were then loaded into the 10X Genomics Chromium Controller, and various libraries (gene expression, TCR, and feature barcoding) were constructed as per manufacturer’s protocol. ICC1 to ICC5 were processed with v1 chemistry (Chromium Single Cell V(D)J Reagent Kits with Feature Barcode technology for Cell Surface Protein) whereas ICC6 and ICC7 were processed with v2 chemistry (Chromium Next GEM Single Cell 5’ Reagent Kits v2 Dual Index with Feature Barcode technology for Cell Surface Protein & Immune Receptor Mapping). Libraries were then sequenced with the NovaSeq 6000.

### Data analyses

10x Genomics Cell Ranger^101^ 5.0.0 was used to build a reference genome index, map reads to a reference genome (GRCh38-2020-A) and quantify genes. Sample-specific hashtags for ICC/IDC-enriched versus benign-enriched cells were demultiplexed by in-house scripts. Briefly, double positive and double negative cells with both or none of ICC/IDC-enriched and benign-enriched hashtags were removed. Whole transcriptome data and hashtag data were stored in the RNA and ADT (Antibody-Derived Tags) assay slots of a S4 Seurat object. A total of 494,151,450 unique reads in 62,995 cells were obtained in 7 patients with an average of 70,593,064 unique reads in 8999 cells per patient. The median and mean of unique reads per cell were 4408 and 7986; the median and mean of unique genes per cell were 1502 and 1945; the median and mean of mitochondrial content per cell were 3.89% and 11.39%. scRNABatchQC^102^ was used to verify consistency and minimal variance of sequenced data based on quality metrics such as unique gene and cell counts across 7 ICC/IDC-enriched and benign-enriched paired samples (Supplementary Fig. 10b–g). The mitochondrial level was assessed by scatter plots of number of reads or number of features (genes) vs. the percentage of mitochondrial content (Supplementary Fig. 10f, g). Overall, 57,697 cells with between 200 and 8000 unique genes, more than 500 unique read counts, and maximum mitochondrial content of 40% were filtered in for further analysis (Supplementary Fig. 10f, g). Seurat^103^ was used for clustering analysis with sctransform based normalization. Cell type of each cluster was initially classified based on cell activity database^104^ and then manually refined based on cell type specific marker gene expression. Marker genes were generated by the FindAllMarkers function of Seurat package with default Wilcoxon Rank Sum test (genes for clusters 0–25 in Source Data). edgeR^105^ was used to detect differential expression across conditions. For each in-cluster and between-cluster differential expression comparison, Counts Per Million values were calculated. The gene with TPM > = 1 in a cell was counted as detected in that cell. The top 10,000 genes detected in a higher number of cells were used in preliminary differential expression analysis, taking into account the ICC/IDC-benign patient pairs by edgeR^51^. Those 10,000 genes were ranked by signaled p-values and used for gene set enrichment analysis by GSEA^106^ package. Only the genes detected in more than 20% cells were used in official differential expression analysis taking into account the Tumor-Benign patient pairs by edgeR (DEG analysis for clusters 0-25 in Source Data). Receptor ligand interactions were inferred using receptor-ligands reported in RIKEN FANTOM5 database^60,107^. Significantly increased DEGs in clusters 5, 6, and 11 (cancer cells) compared to cluster 12 (benign epithelial cells) were compared to ligands reported in RIKEN FANTOM5 database. The expression of corresponding receptors was assessed in cluster 20 cells. Monocle 3 v1.0.1 package^108,109^ was used for pseudotime analyses. 10x Genomics Cell Ranger and enclone were used for V(D)J T cell analysis. Briefly, the CellRanger files (all_contig_annotations.json) from different samples were combined to one all_contig_annotations.json file. Then enclone was used to find and organize cells arising from the same progenitors into groups (clonotypes). The default code allowed for clonotypes with 2 alpha plus 1 beta chain or 1 alpha plus 2 beta chains. Approximately 3% (281 out of 9398) of the clonotypes assayed had two beta chains. As a default, cells that expressed more than 4 productive chains were removed from the output. Finally, the top 20 clonotypes with most cells were visualized in gene expression based UMAP. A clonotype was considered enriched if its frequency in ICC/IDC-enriched prostate was at least double of the matching frequency in benign-enriched prostate or had ≥2 cells if present only in ICC/IDC-enriched prostate. Similarly, a clonotype was considered contracted if its frequency in ICC/IDC-enriched prostate was half or less of the matching frequency in the benign-enriched prostate or had ≥2 cells if present only in benign-enriched prostate. Simpson clonality was used to determine T cell clonotype evenness. Simpson clonality was calculated as the square root of the Simpson’s index, which is the summation of the square of proportional abundance of all observed clonotypes^110^. The proportional abundance of a clonotype is the number of T cells of that clonotype divided by the total number of T cells with assigned clonotypes. R packages ggplot2 and reshape2 were used to graph dimplots, featureplots, and violin plots; R packages tidyverse and pheatmap were used to plot heatmaps; Reclustering was performed with R packages patchwork, kableExtra, and dplyr. Scripts for analysis and visualization were deposited on GitHub.

### Immunohistochemistry

Patient formalin-fixed paraffin-embedded (FFPE) whole tissue sections (4 µm) from RP specimens were analyzed by immunohistochemistry. Slides for anti-PTEN, anti-CD31, anti-AR, and anti-MYC were placed on the Leica Bond-RX for IHC staining. All steps besides dehydration, clearing and cover slipping were performed on the Bond-RX. Slides were deparaffinized. Heat-induced antigen retrieval was performed on the Bond-RX using their Epitope Retrieval 2 solution. Slides were incubated with anti-PTEN (DAKO; #M3627; dilution 1:250), anti-CD31 (Leica; #PA0250; dilution Ready to Use), anti-AR (Roche; #760-4605; clone SP107, dilution Ready to Use), or anti-cMYC (Abcam; #ab32072; dilution 1:100). The Bond Polymer Refine detection system was used for visualization. Slides were then dehydrated, cleared, and cover slipped. Antibodies directed against PTEN, CD31, AR, and MYC were previously validated for IHC by the VUMC translational pathology shared resource using known human positive and negative control tissue. Experimental IHC slides had control slides included. The study pathologist confirmed the accuracy of the antibody staining. Slides for anti-ERG and ProsC were performed on the Leica Bond III. Epitope Retrieval 1 was used for anti-ERG (Biocare Medical; #PM421AA; dilution Ready to Use) and Epitope Retrieval 2 was used for ProsC (CK HMW^+^, TP63^+^, AMACR; Biocare Medical; #API3154DSAA; dilution Ready to Use) both for 20 minutes pretreatment and 15 minutes antibody retrieval. PTEN loss, ERG positivity as defined by diffuse nuclear staining, and AR positivity as defined by diffuse nuclear staining were scored in comparison to included control tissue and benign-adjacent tissue. Antibodies directed against ERG and ProsC were previously validated for IHC by the VUMC clinical pathology laboratory. Antibodies were validated on 10 known human positive cases and 10 known human negative cases. The medical director reviewed and confirmed the accuracy of the antibody staining. Experimental IHC slides had control tissue included.

### Dual RNA in situ hybridization

ICC1-7 and an extended cohort of patient FFPE whole tissue sections (4 µm) from RP specimens were analyzed for *SCHLAP1* (ACD; #534271) and *JAG1* (ACD; #546181-C2) using the RNAscope® 2.5 HD Duplex Assay by Advanced Cell Diagnostics (ACD; #322430) according to the manufacturer’s recommendations. *SCHLAP1* and *JAG1* expression were assessed by H-score: (3 × % strong stain) + (2 × % moderate stain)  + (1 x % weak stain). *SCHLAP1* and *JAG1* expression were assessed by a pathologist in benign prostate and in Gleason pattern 3, Gleason pattern 4 non-ICC, ICC/IDC, and Gleason pattern 5 prostate cancer. An extended cohort of patient FFPE whole tissue sections (4 µm) from RP specimens were analyzed for *KRT13* (ACD, #843401) and *SCGB1A1* (ACD; #469971-C2) using the RNAscope® 2.5 HD Duplex Assay by Advanced Cell Diagnostics (ACD; #322430) according to the manufacturer’s recommendations. *KRT13* and *SCGB1A1* expression was assessed by a pathologist in benign prostate and in Gleason pattern, Gleason pattern 4 non-ICC, ICC/IDC, and Gleason pattern 5 prostate cancer. Patient FFPE whole tissue sections (4 µm) from RP specimens were analyzed for *CTHRC1* (ACD; #413331), *ASPN* (ACD; #404481), *FAP* (ACD; #411971) and *ENG* (ACD; #484111) using the RNAscope® 2.5 HD Duplex Assay by Advanced Cell Diagnostics (ACD; #322430) according to the manufacturer’s recommendations. *CTHRC1, ASPN, FAP*, and *ENG* expressions were assessed using Halo Software. Whole slides were scanned for brightfield imaging at the Vanderbilt Digital Histology Core (SCN400; Leica, Wetzlar, Germany). Representative areas of stroma adjacent to predominantly benign prostate as well as Gleason pattern 3, Gleason pattern 4 non-ICC, ICC/IDC, and Gleason pattern 5 prostate cancer were identified by a pathologist, and probe staining was analyzed using Halo Software v.3.4.2986 (new cases) or v3.0.311.328 (historical cases/analyses) (Indica Labs, Albuquerque, NM, USA). Slides were quantified for the percentage of stromal cells that were positive for the probe. Intensity of probe staining was quantified and then divided by the number of positive stromal cells to determine the relative mean expression per positive stromal cell. The expression score was calculated by the percent positive stromal cells x the relative mean expression per positive stromal cells. New cases were examined both independently and in combination with available historical cases^33^.

### TCGA and MSKCC data

Prostate Adenocarcinoma (TCGA, PanCancer Atlas) data^74^ and MSKCC Prostate Adenocarcinoma data^75^ were obtained from cBioPortal^111,112^. Core survival analysis based on collated Z-scores was performed using R package survival, and Kaplan-Meier survival curves were plotted with R packages survminer and ggplot2. An initial 8 gene signature (*TGFB1, TNC*, *SFRP4, CCL2*, *CTHRC1, ASPN, FAP*, and *ENG*) was weighted evenly between the eight genes and then examined by median Z-score expression and log-rank test for PFS as determined by the period from the date of diagnosis until the date of the first occurrence of a new tumor event (NTE), which includes a progression of the disease, locoregional recurrence, distant metastasis, new primary tumor, or death with tumor (*n* = 492). The 8 gene signature was also examined by median Z-score expression in primary tumors in the MSKCC Prostate Adenocarcinoma cohort for Disease Free survival as defined by biochemical recurrence (PSA ≥ 0.2 ng/mL on two occasions) (*n* = 131) (Supplementary Fig. 6b). Individual genes in the 8 gene signature were also examined for associations using the same methods in both cohorts. Only individual genes that had a significant association with worse outcomes in both cohorts were included in the final signature and assessed for further examination by RNAscope (*CTHRC1, ASPN, FAP*, and *ENG*). The CAFÉ CAF 4 gene signature *(CTHRC1, ASPN, FAP, and ENG)* was analyzed using the same methods above as the 8 gene signature. In addition, a *C1QB*^*+*^*TREM2*^*+*^*APOE*^*+*^ M2 Signature (*C1QB, TREM2, APOE, CD163, MSR1, MRC1*) was analyzed in the TCGA Prostate Adenocarcinoma and MSKCC Prostate Adenocarcinoma cohorts using the same metrics.

### Statistical analyses

Unless otherwise indicated, statistical comparisons between two groups were performed using a two-tailed Student *t* test, with specifications indicated in the figure legends. Statistical comparisons between multiple groups were performed using one-way ANOVA with Tukey’s multiple comparisons, as indicated in the figure legends. Statistical significance was defined as a *P* < 0.05 and exact *P* values were indicated in the figures. Statistical comparisons were performed using GraphPad Prism software (v5.0) or Seurat V3^113^ in R Studio.

### Reporting summary

Further information on research design is available in the Nature Research Reporting Summary linked to this article.

## Supplementary information

Supplementary Information

Reporting Summary

## Data Availability

The publicly available Prostate Adenocarcinoma (TCGA, PanCancer Atlas) data^74^ and MSKCC Prostate Adenocarcinoma data^75^ are available from cBioPortal^111,112^ https://www.cbioportal.org/datasets. The RIKEN FANTOM5 database is publicly available: https://fantom.gsc.riken.jp/5/. The single-cell RNA-sequencing data generated in this study have been deposited in the Gene Expression Omnibus (GEO) database under accession code GSE185344: Source data are provided in this paper as a Source data file. The remaining data are available within the Article, Supplementary Information, and Source Data file. Source data are provided with this paper.

## Source data

Source Data

## Code availability

Code can be found on GitHub: https://github.com/shengqh/Hurley2022scRNA/.
