# Supplementary material for: Single cell analysis of cribriform prostate cancer reveals cell intrinsic and tumor microenvironmental pathways of aggressive disease
Source: Nat Commun. 2022 Oct 13;13:6036. doi: 10.1038/s41467-022-33780-1 (PMC9562361; doi:10.1038/s41467-022-33780-1)
Supplement: Supplementary file 1 — Supplementary Information [file 41467_2022_33780_MOESM1_ESM.pdf]

Supplementary Information

Single Cell Analysis of Cribriform Prostate Cancer Reveals Cell Intrinsic and Tumor Microenvironmental Pathways of Aggressive Disease

Authors: Hong Yuen Wong[1], Quanhu Sheng[2], Amanda B. Hesterberg[1], Sarah Croessmann[1], Brenda L. Rios[1], Khem Giri[1], Jorgen Jackson[1], Adam X. Miranda[1], Evan Watkins[1], Kerry R. Schaffer[1,3], Meredith Donahue[4], Elizabeth Winkler[4], David F. Penson[3,4], Joseph A. Smith[4], S. Duke Herrell[4], Amy N. Luckenbaugh[4], Daniel A. Barocas[4], Young J. Kim[3,5,6], Diana Graves[7], Giovanna A. Giannico[7], Jeffrey C. Rathmell[3,7,8], Ben H. Park[1,3], Jennifer B. Gordetsky[3,7], Paula J. Hurley[1,3,4]

Author Affiliations:

[1]Department of Medicine, Vanderbilt University Medical Center, Nashville, TN, USA

[2]Department of Biostatistics, Vanderbilt University Medical Center, Nashville, TN, USA

[3]Vanderbilt-Ingram Cancer Center, Nashville, TN, USA

[4]Department of Urology, Vanderbilt University Medical Center, Nashville, TN, USA

[5]Department of Otolaryngology-Head and Neck Surgery, Vanderbilt University Medical Center, Nashville, TN, USA

[6]Regeneron Pharmaceuticals, Tarrytown, New York, USA

[7]Department of Pathology, Microbiology, and Immunology, Vanderbilt University Medical Center, Nashville, TN, USA

[8]Vanderbilt Center for Immunobiology, Nashville, TN, USA

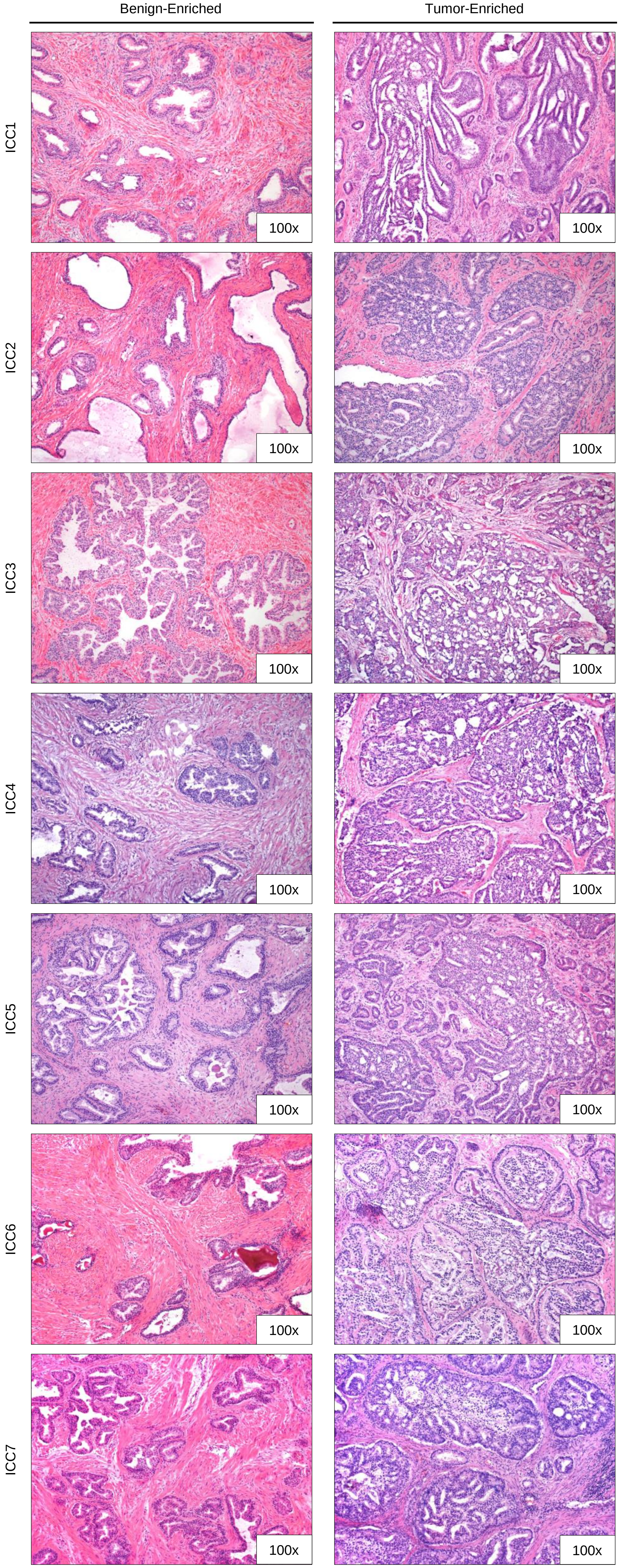

Benign-Enriched
Tumor-Enriched
ICC1
ICC2
ICC3
ICC4
ICC5
ICC6
ICC7
100x

Supplementary Figure 1. Rapid frozen H&Es of benign-enriched and ICC/IDC-enriched prostate isolated for scRNAseq. H&Es shown per patient at 100x, bar = 200 µm, (ICC1-7; n=7 biologically independent samples). Subset of images also shown in Fig. 1b.

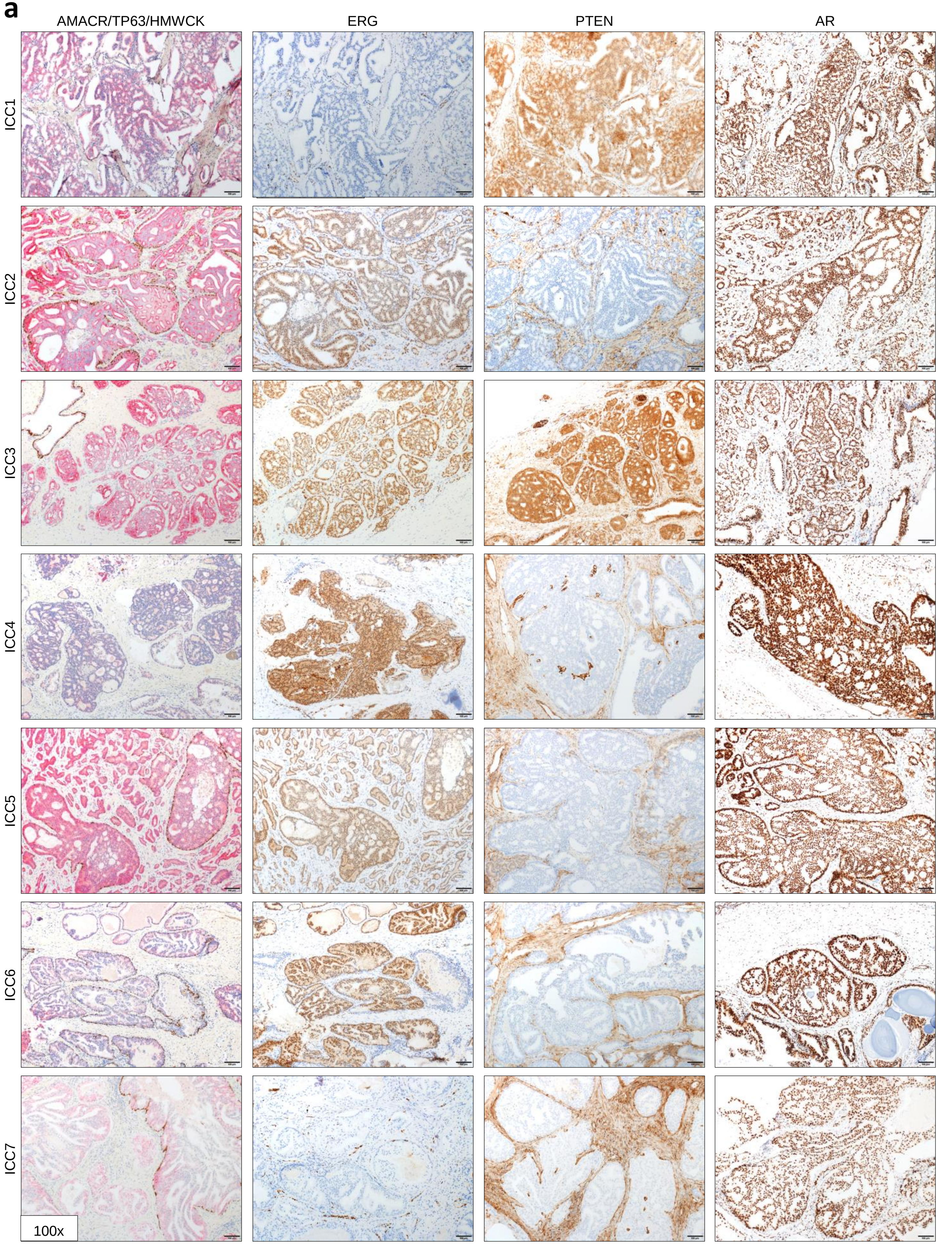
a
AMACR/TP63/HMWCK
ERG
PTEN
AR
ICC1
ICC2
ICC3
ICC4
ICC5
ICC6
ICC7
100x

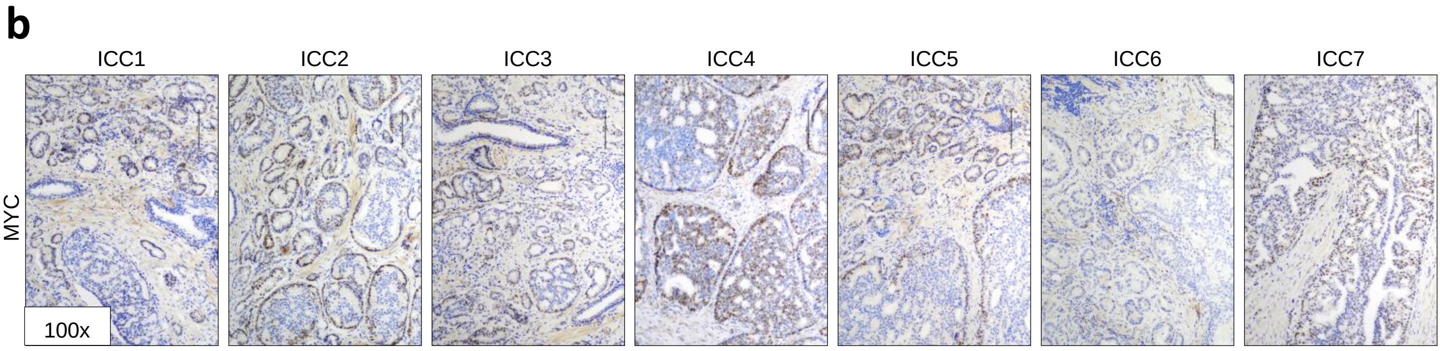
b
ICC1
ICC2
ICC3
ICC4
ICC5
ICC6
ICC7
MYC
100x

Supplementary Figure 2. Protein marker analysis of ICC/IDC-enriched prostate. a. AMACR, TP63, HMWCK, ERG, PTEN, and AR staining by IHC on FFPE prostate tissue from RP at 100x, bar = 100 µm, (ICC1-7; n=7 biologically independent samples). Subset of images also shown in Fig. 1d. **b.** MYC staining by IHC on FFPE prostate tissue sections from RP at 100x, bar = 100 µm, (ICC1-7; n=7 biologically independent samples).

a

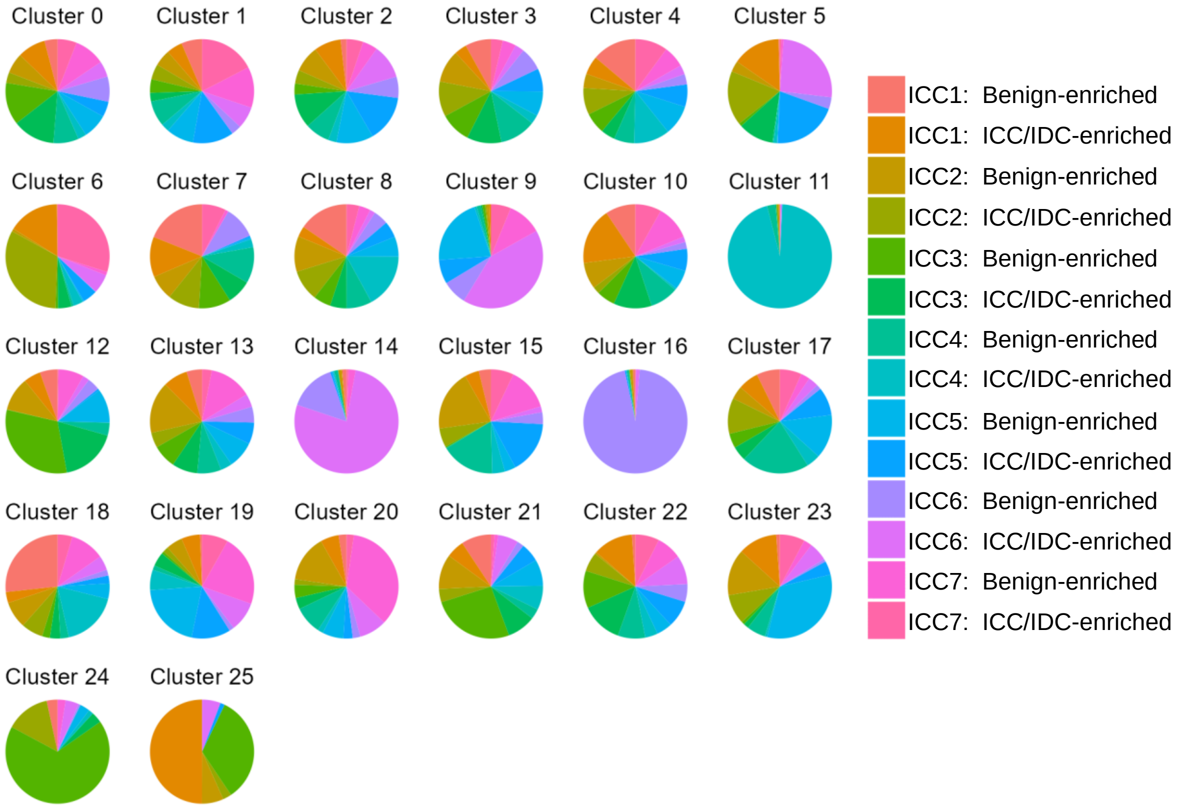
Cluster 0
Cluster 1
Cluster 2
Cluster 3
Cluster 4
Cluster 5
Cluster 6
Cluster 7
Cluster 8
Cluster 9
Cluster 10
Cluster 11
Cluster 12
Cluster 13
Cluster 14
Cluster 15
Cluster 16
Cluster 17
Cluster 18
Cluster 19
Cluster 20
Cluster 21
Cluster 22
Cluster 23
Cluster 24
Cluster 25
ICC1: Benign-enriched
ICC1: ICC/IDC-enriched
ICC2: Benign-enriched
ICC2: ICC/IDC-enriched
ICC3: Benign-enriched
ICC3: ICC/IDC-enriched
ICC4: Benign-enriched
ICC4: ICC/IDC-enriched
ICC5: Benign-enriched
ICC5: ICC/IDC-enriched
ICC6: Benign-enriched
ICC6: ICC/IDC-enriched
ICC7: Benign-enriched
ICC7: ICC/IDC-enriched

b

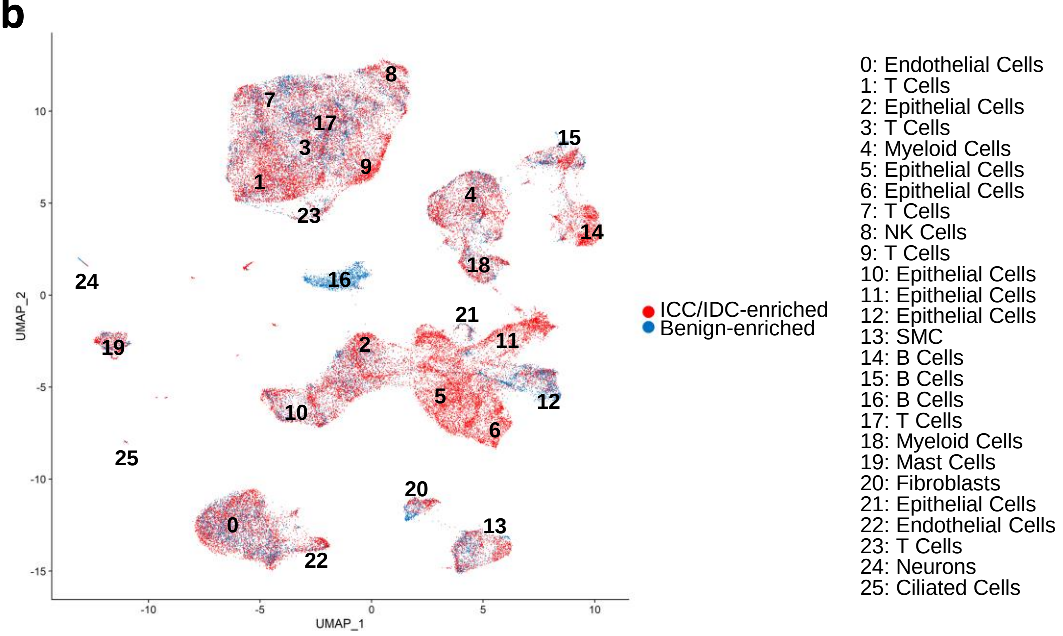
UMAP_1
UMAP_2
ICC/IDC-enriched
Benign-enriched
0: Endothelial Cells
1: T Cells
2: Epithelial Cells
3: T Cells
4: Myeloid Cells
5: Epithelial Cells
6: Epithelial Cells
7: T Cells
8: NK Cells
9: T Cells
10: Epithelial Cells
11: Epithelial Cells
12: Epithelial Cells
13: SMC
14: B Cells
15: B Cells
16: B Cells
17: T Cells
18: Myeloid Cells
19: Mast Cells
20: Fibroblasts
21: Epithelial Cells
22: Endothelial Cells
23: T Cells
24: Neurons
25: Ciliated Cells

Supplementary Figure 3. The contribution of ICC/IDC-enriched and benign-enriched cells to each cluster. **a.** Percentage of cells per sample in each cluster (ICC1-7; n=7 biologically independent samples). Total cells in each sample were normalized to equal number. **b.** Unsupervised graph-based clustering of all samples visualized by UMAP delineated by benign-enriched and ICC/IDC-enriched prostate (ICC1-7; n=7 biologically independent samples).

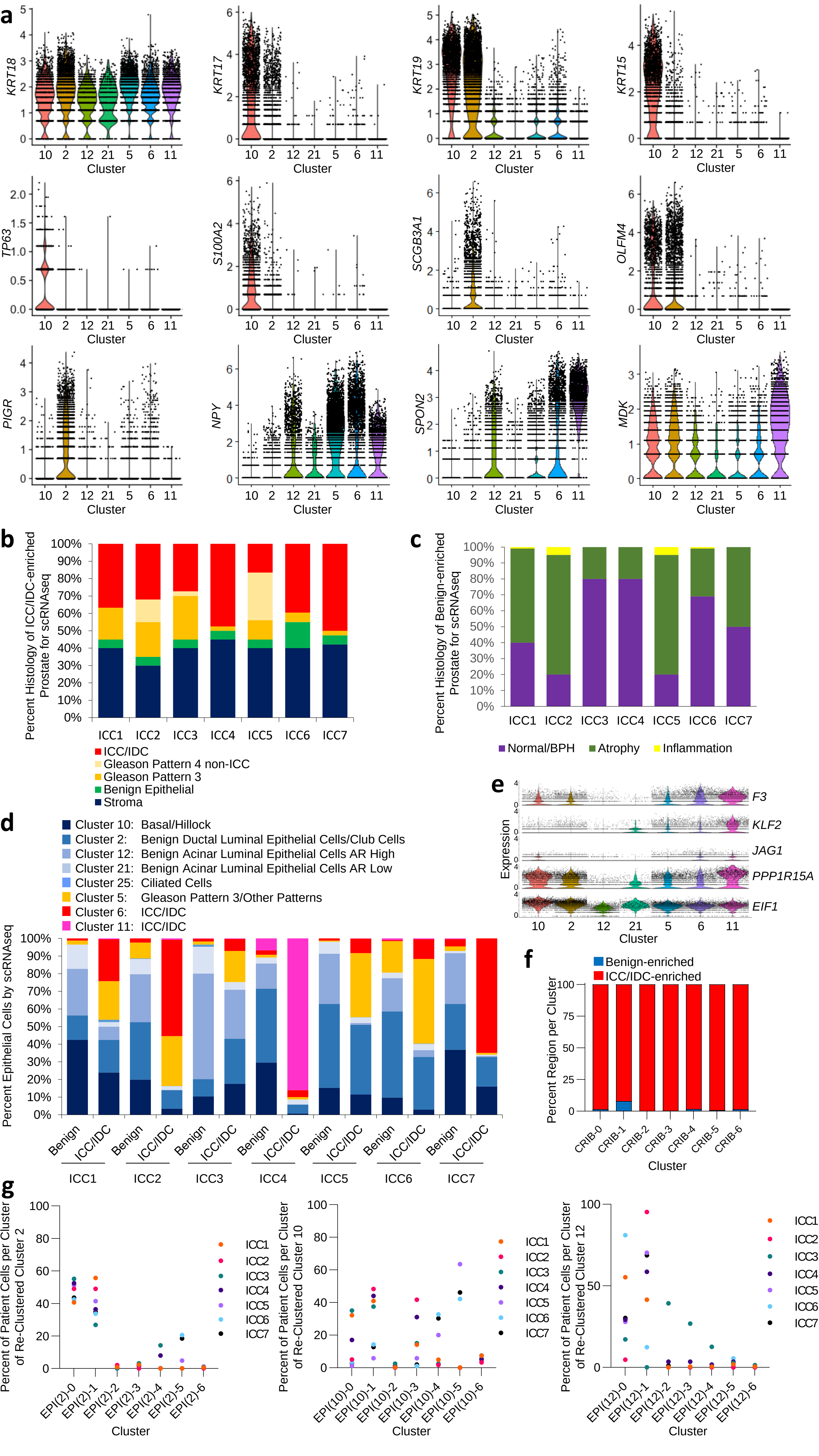
a
KRT18
KRT17
KRT19
KRT15
TP63
S100A2
SCGB3A1
OLFM4
PIGR
NPY
SPON2
MDK
10 2 12 21 5 6 11
Cluster
b
Percent Histology of ICC/IDC-enriched Prostate for scRNAseq
ICC1 ICC2 ICC3 ICC4 ICC5 ICC6 ICC7
ICC/IDC
Gleason Pattern 4 non-ICC
Gleason Pattern 3
Benign Epithelial
Stroma
c
Percent Histology of Benign-enriched Prostate for scRNAseq
Normal/BPH
Atrophy
Inflammation
d
Cluster 10: Basal/Hillock
Cluster 2: Benign Ductal Luminal Epithelial Cells/Club Cells
Cluster 12: Benign Acinar Luminal Epithelial Cells AR High
Cluster 21: Benign Acinar Luminal Epithelial Cells AR Low
Cluster 25: Ciliated Cells
Cluster 5: Gleason Pattern 3/Other Patterns
Cluster 6: ICC/IDC
Cluster 11: ICC/IDC
Percent Epithelial Cells by scRNAseq
Benign ICC/IDC
e
Expression
F3
KLF2
JAG1
PPP1R15A
EIF1
f
Benign-enriched
ICC/IDC-enriched
Percent Region per Cluster
CRIB-0 CRIB-1 CRIB-2 CRIB-3 CRIB-4 CRIB-5 CRIB-6
g
Percent of Patient Cells per Cluster of Re-Clustered Cluster 2
EPI(2)-0 EPI(2)-1 EPI(2)-2 EPI(2)-3 EPI(2)-4 EPI(2)-5 EPI(2)-6
Percent of Patient Cells per Cluster of Re-Clustered Cluster 10
EPI(10)-0 EPI(10)-1 EPI(10)-2 EPI(10)-3 EPI(10)-4 EPI(10)-5 EPI(10)-6
Percent of Patient Cells per Cluster of Re-Clustered Cluster 12
EPI(12)-0 EPI(12)-1 EPI(12)-2 EPI(12)-3 EPI(12)-4 EPI(12)-5 EPI(12)-6

Supplementary Figure 4. Epithelial cell marker expression and histology analysis. a. Violin plots of markers in epithelial clusters 10, 2, 12, 21, 5, 6, and 11. **b.** Percent histology of ICC/IDC-enriched prostate isolated for scRNAseq. **c.** Percent histology of benign-enriched prostate isolated for scRNAseq. **d**. Percent of epithelial cell clusters in benign-enriched and ICC/IDC-enriched prostate by scRNAseq. **e.** Violin plots of the top 5 ranked TNFα signaling via NFκB hallmark genes by pssGSEA between ICC/IDC cells in cluster 6 and benign epithelial cells in cluster 12. **f.** The percent of benign-enriched and ICC/IDC-enriched cells contributing to re-clustered clusters 5, 6, and 11 into CRIB-0-6. **g**. The percent of patient cells per cluster after individual re-clustering of clusters 2, 10 and 12 into 7 clusters each. Source data are provided as a Source Data file.

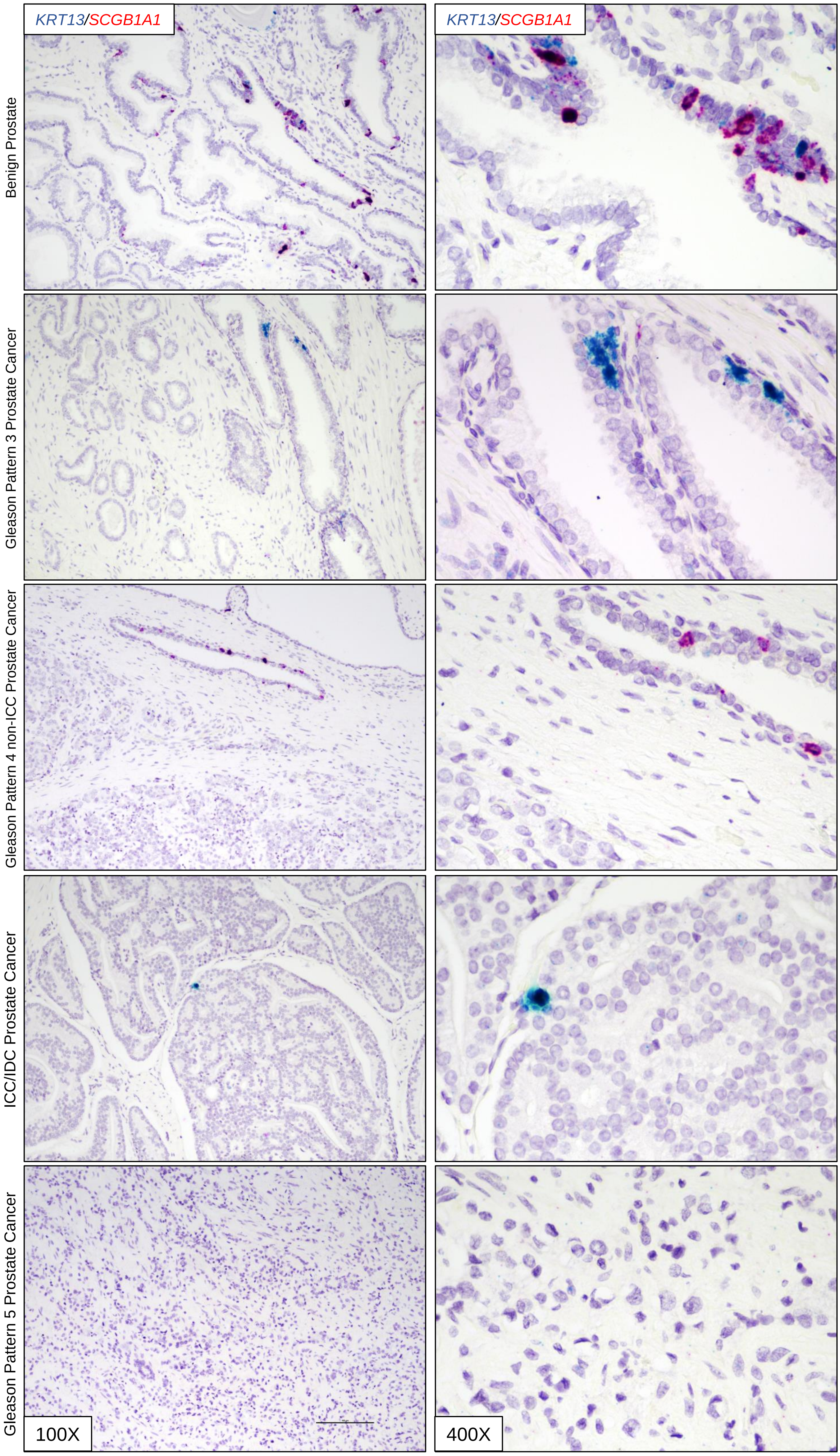
KRT13/SCGB1A1
KRT13/SCGB1A1
Benign Prostate
Gleason Pattern 3 Prostate Cancer
Gleason Pattern 4 non-ICC Prostate Cancer
ICC/IDC Prostate Cancer
Gleason Pattern 5 Prostate Cancer
100X
400X

Supplementary Figure 5. Hillock and club cell marker expression in benign prostate and prostate cancer by grade and histology. Representative images of *KRT13* (blue) and *SCGB1A1* (red) by RNAscope on FFPE prostate tissue from RP in benign prostate (n=24) and in Gleason pattern 3 (n=18), Gleason pattern 4 non-ICC (n=9), ICC/IDC (n=9), and Gleason pattern 5 (n=6) prostate cancer at 100x (bar = 100 µm) and 400x. A total of n=24 biologically independent samples were used to assess *KRT13* and *SCGB1A1* expression with samples having more than one histology for assessment.

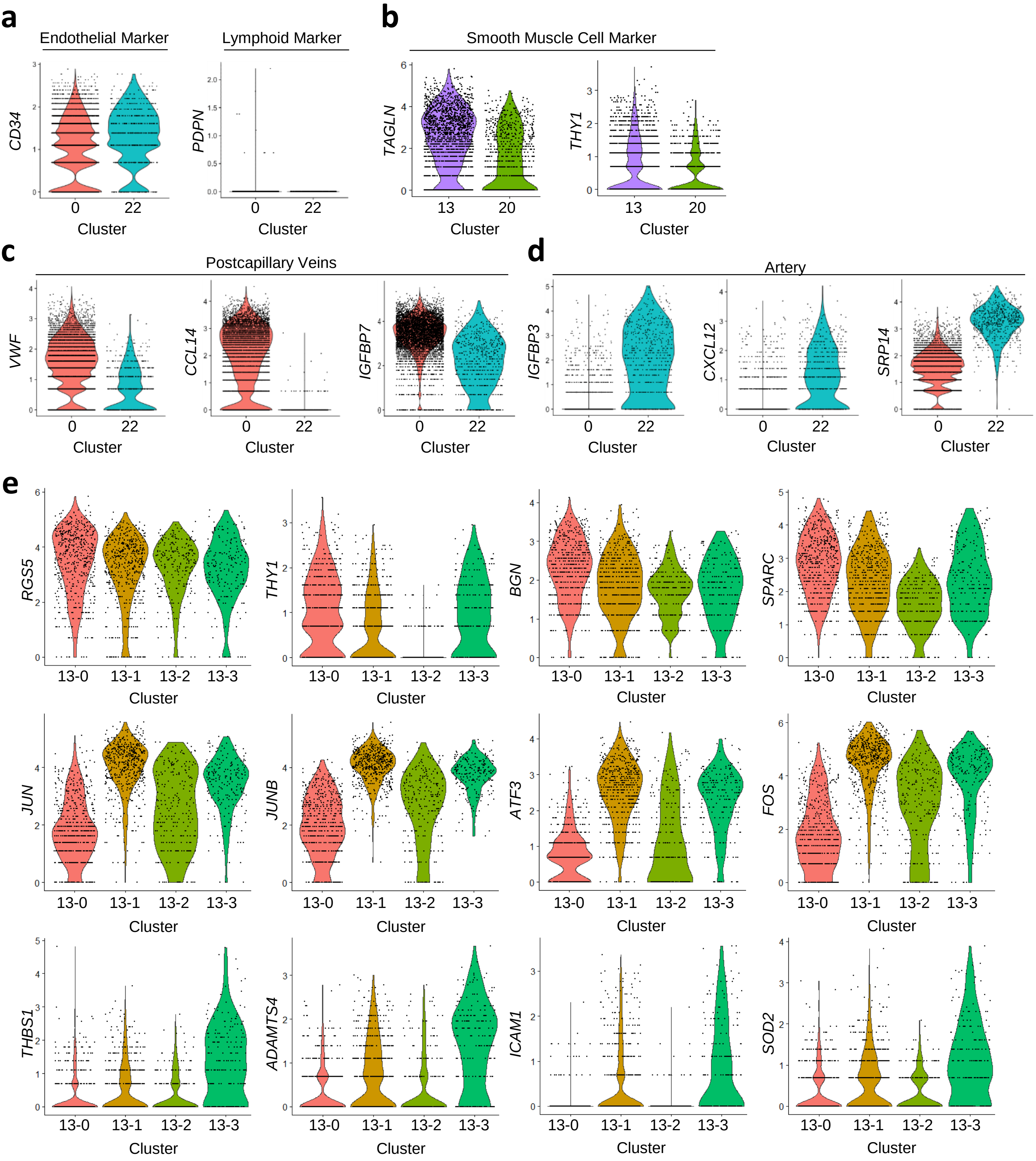

a
Endothelial Marker
Lymphoid Marker
CD34
PDPN
b
Smooth Muscle Cell Marker
TAGLN
THY1
c
Postcapillary Veins
VWF
CCL14
IGFBP7
d
Artery
IGFBP3
CXCL12
SRP14
e
RGS5
THY1
BGN
SPARC
JUN
JUNB
ATF3
FOS
THBS1
ADAMTS4
ICAM1
SOD2
Cluster
0
22
13
20
13-0
13-1
13-2
13-3

Supplementary Figure 6. Smooth muscle cell gene expression. a. Violin plots of endothelial and lymphoid marker expression in clusters 0 and 22. **b.** Violin plots of SMC markers in clusters 13 and 20. **c, d.** Violin plots of endothelial cell markers in clusters 0 and 22. **e.** Violin plots of markers in clusters 13-0 through 13-3.

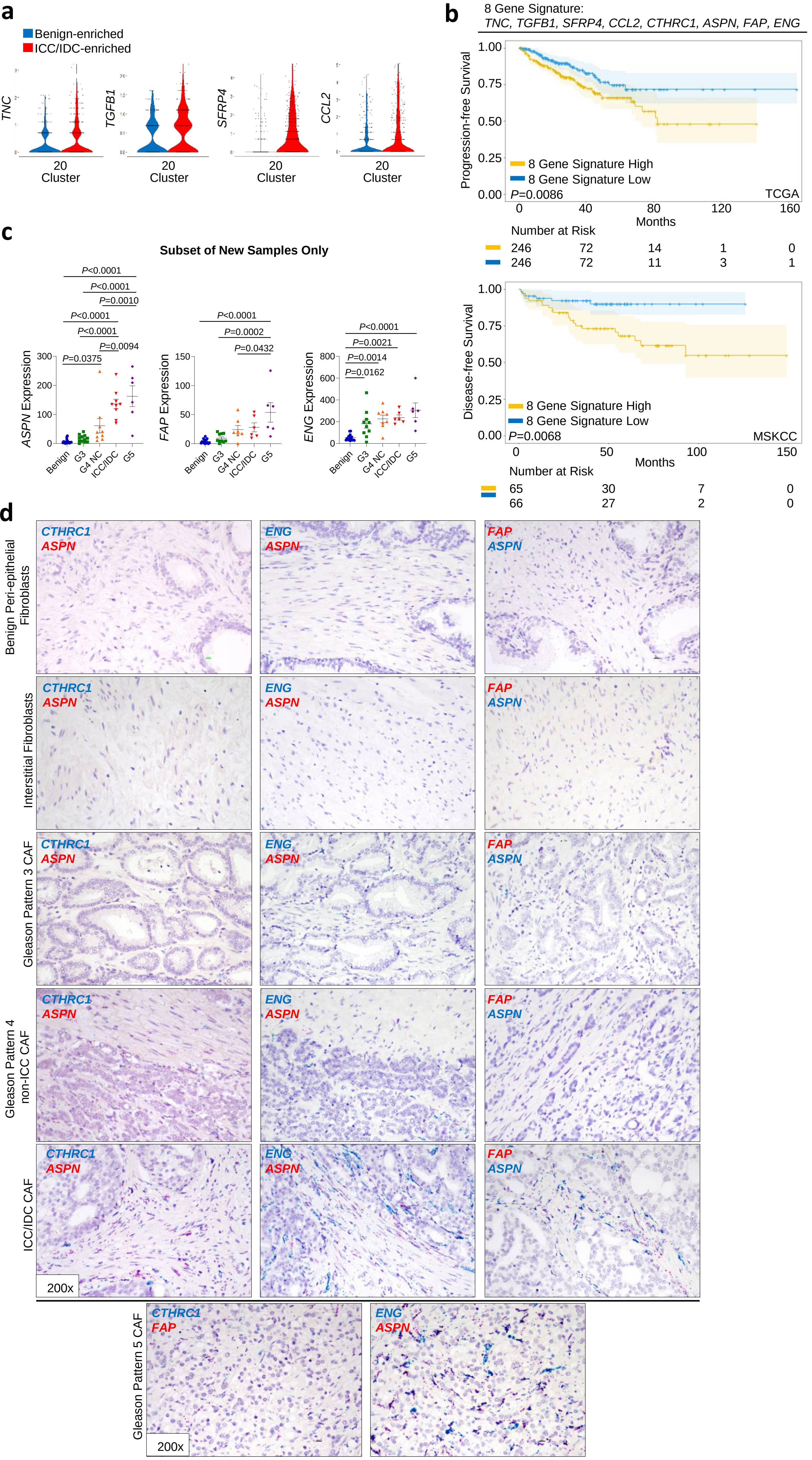
a
Benign-enriched
ICC/IDC-enriched
TNC
TGFB1
SFRP4
CCL2
20
Cluster
b
8 Gene Signature:
TNC, TGFB1, SFRP4, CCL2, CTHRC1, ASPN, FAP, ENG
Progression-free Survival
8 Gene Signature High
8 Gene Signature Low
P=0.0086
TCGA
Months
Number at Risk
246 72 14 1 0
246 72 11 3 1
Disease-free Survival
8 Gene Signature High
8 Gene Signature Low
P=0.0068
MSKCC
Months
Number at Risk
65 30 7 0
66 27 2 0
c
Subset of New Samples Only
ASPN Expression
P<0.0001
P<0.0001
P=0.0010
P<0.0001
P<0.0001
P=0.0094
P=0.0375
FAP Expression
P<0.0001
P=0.0002
P=0.0432
ENG Expression
P<0.0001
P=0.0021
P=0.0014
P=0.0162
Benign
G3
G4 NC
ICC/IDC
G5
d
Benign Peri-epithelial Fibroblasts
Interstitial Fibroblasts
Gleason Pattern 3 CAF
Gleason Pattern 4 non-ICC CAF
ICC/IDC CAF
Gleason Pattern 5 CAF
CTHRC1
ASPN
ENG
FAP
200x

Supplementary Figure 7. CAFÉ CAF expression in benign prostate and prostate cancer by grade and histology. a. Violin plots of markers differentially expressed in cluster 20 between ICC/IDC-enriched prostate and benign-enriched prostate**. b.** Kaplan-Meier and log-rank test of progression-free survival in the TCGA PanCancer Atlas Prostate Adenocarcinoma for the 8 gene CAF signature (*TNC, TGFB1, SFRP4, CCL2, CTHRC1, ASPN, FAP*, and *ENG*) by median expression (n=492). Kaplan-Meier and log-rank test of disease-free survival (DFS) in the MSKCC Prostate Adenocarcinoma for the 8 gene CAF signature by median expression (n=131). **c.** Quantification of expression (intensity x percent) of *ASPN*, *FAP*, and *ENG* by RNAScope and Halo Software in only the new subset of the extended RP prostate cancer cohort adjacent to benign prostate (*ASPN* n=19, *FAP* n=16, and *ENG* n=16), Gleason pattern 3 (G3; *ASPN* n=12, *FAP* n=10, and *ENG* n=10), Gleason pattern 4 non-ICC (G4 NC; *ASPN* n=9, *FAP* n=7, and *ENG* n=8), ICC/IDCC (*ASPN* n=9, *FAP* n=6, and *ENG* n=6), and Gleason pattern 5 (G5; *ASPN* n=6, *FAP* n=6, and *ENG* n=6) prostate cancer. A total of n=21 biologically independent samples were used to assess *CTHRC1*, *ASPN*, *FAP*, and *ENG* expression with samples having more than one histology for assessment and sample overlap between markers. Graphs shown as mean ± SEM and analyzed by one-way Anova with Tukey's Multiple Comparisons. Quantification of *CTHRC1* expression is shown in Fig. 5m as only new samples were available for *CTHRC1*. **d.** Corresponding representative images of *CTHRC1, ASPN, FAP,* and *ENG* by RNAscope as quantified in Supplementary Fig. 7c in fibroblasts peri-epithelial to benign prostate, interstitial fibroblasts as well as CAF adjacent to Gleason pattern 3, Gleason pattern 4 non-ICC, ICC/IDC, and Gleason pattern 5 prostate cancer from RP at 200x, bar = 10 µm. Source data are provided as a Source Data file.

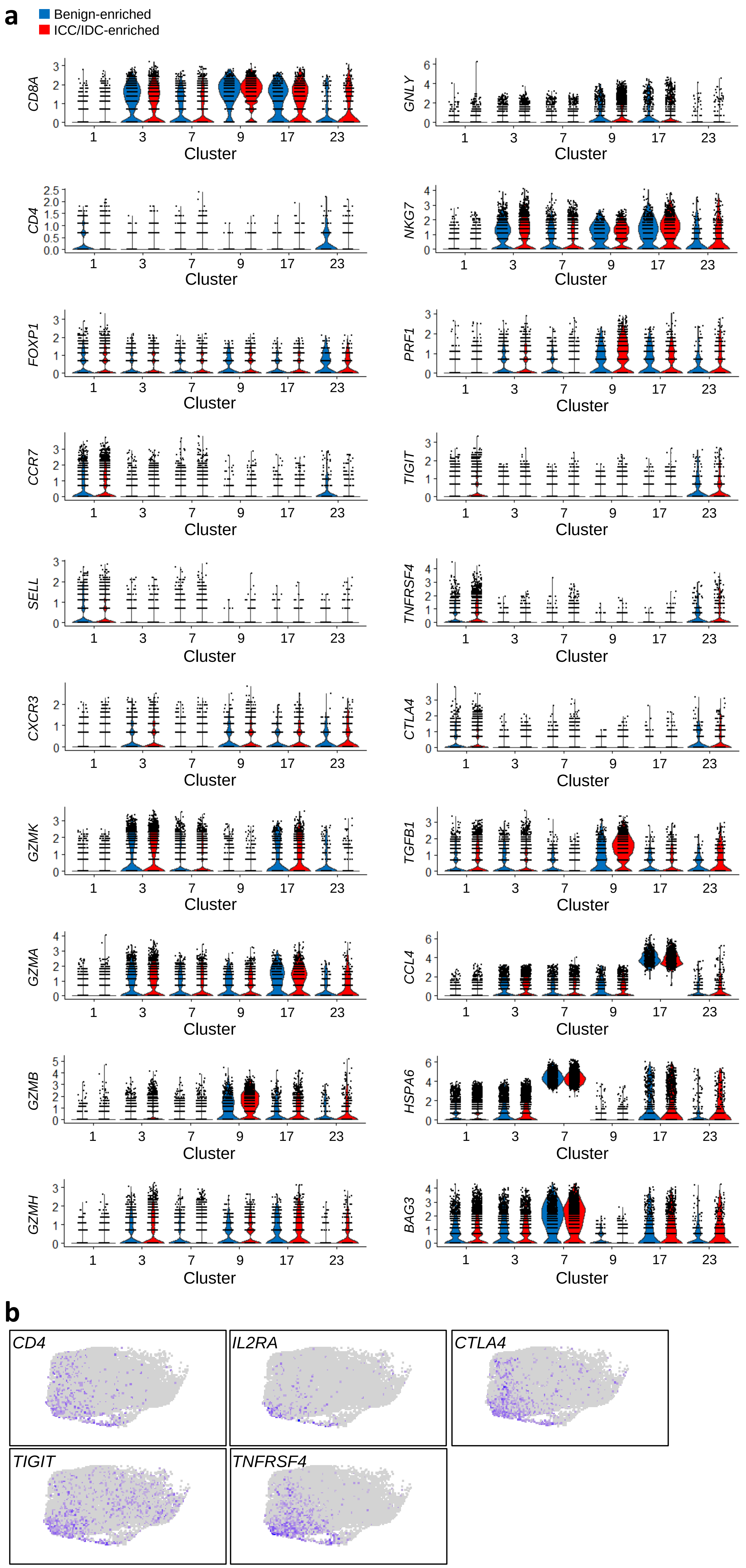
a
Benign-enriched
ICC/IDC-enriched
CD8A
GNLY
CD4
NKG7
FOXP1
PRF1
CCR7
TIGIT
SELL
TNFRSF4
CXCR3
CTLA4
GZMK
TGFB1
GZMA
CCL4
GZMB
HSPA6
GZMH
BAG3
Cluster
1
3
7
9
17
23
b
CD4
IL2RA
CTLA4
TIGIT
TNFRSF4

Supplementary Figure 8. T cell gene expression. a. Violin plots of markers in ICC/IDC-enriched prostate compared to benign-enriched prostate in clusters 1, 3, 7, 9, 17, and 23. **b**. Feature plots of markers expressed in T cell clusters from unsupervised graph-based clustering.

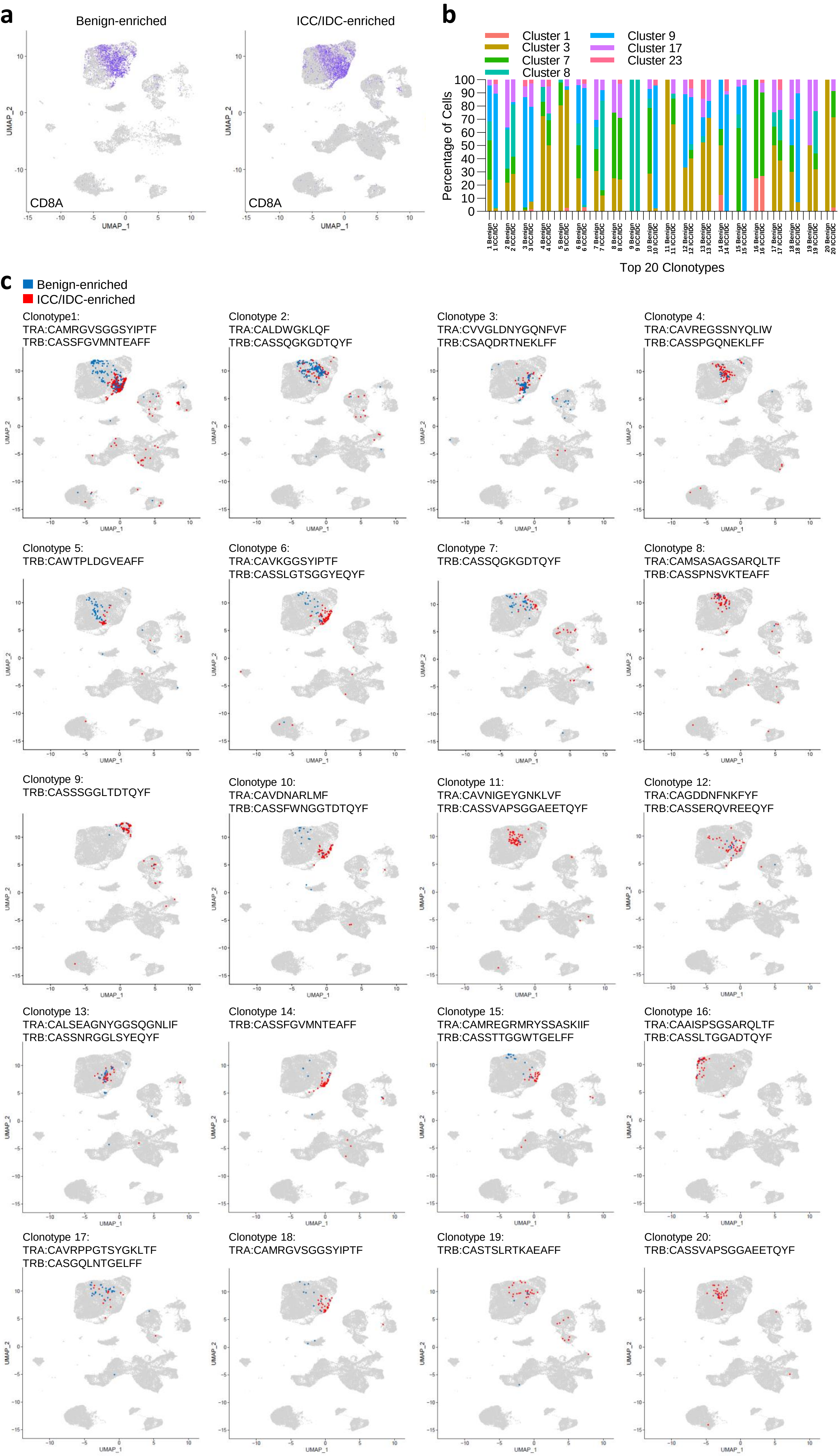
a
Benign-enriched
ICC/IDC-enriched
CD8A
CD8A
UMAP_1
UMAP_2
b
Cluster 1
Cluster 3
Cluster 7
Cluster 8
Cluster 9
Cluster 17
Cluster 23
Percentage of Cells
Top 20 Clonotypes
c
Benign-enriched
ICC/IDC-enriched
Clonotype1:
TRA:CAMRGVSGGSYIPTF
TRB:CASSFGVMNTEAFF
Clonotype 2:
TRA:CALDWGKLQF
TRB:CASSQGKGDTQYF
Clonotype 3:
TRA:CVVGLDNYGQNFVF
TRB:CSAQDRTNEKLFF
Clonotype 4:
TRA:CAVREGSSNYQLIW
TRB:CASSPGQNEKLFF
Clonotype 5:
TRB:CAWTPLDGVEAFF
Clonotype 6:
TRA:CAVKGGSYIPTF
TRB:CASSLGTSGGYEQYF
Clonotype 7:
TRB:CASSQGKGDTQYF
Clonotype 8:
TRA:CAMSASAGSARQLTF
TRB:CASSPNSVKTEAFF
Clonotype 9:
TRB:CASSSGGLTDTQYF
Clonotype 10:
TRA:CAVDNARLMF
TRB:CASSFWNGGTDTQYF
Clonotype 11:
TRA:CAVNIGEYGNKLVF
TRB:CASSVAPSGGAEETQYF
Clonotype 12:
TRA:CAGDDNFNKFYF
TRB:CASSERQVREEQYF
Clonotype 13:
TRA:CALSEAGNYGGSQGNLIF
TRB:CASSNRGGLSYEQYF
Clonotype 14:
TRB:CASSFGVMNTEAFF
Clonotype 15:
TRA:CAMREGRMRYSSASKIIF
TRB:CASSTTGGWTGELFF
Clonotype 16:
TRA:CAAISPSGSARQLTF
TRB:CASSLTGGADTQYF
Clonotype 17:
TRA:CAVRPPGTSYGKLTF
TRB:CASGQLNTGELFF
Clonotype 18:
TRA:CAMRGVSGGSYIPTF
Clonotype 19:
TRB:CASTSLRTKAEAFF
Clonotype 20:
TRB:CASSVAPSGGAEETQYF

Supplementary Figure 9. Mapping of the top twenty TCR clonotypes in benign-enriched prostate and ICC/IDC-enriched prostate cancer. a. Feature plots of CD8A on ICC/IDC-enriched prostate compared to benign-enriched prostate in clusters 1, 3, 7, 9, 17, and 23. **b.** The percentage of the cells from the top 20 clonotypes in clusters 1, 3, 7, 8, 9, 17, and 23 separated by benign-enriched prostate and ICC/IDC-enriched prostate cancer. **c.** Clonotype mapping of the top twenty TCR clonotypes in ICC/IDC-enriched prostate compared to benign-enriched prostate in clusters 1, 3, 7, 9, 17, and 23. Source data are provided as a Source Data file.

a

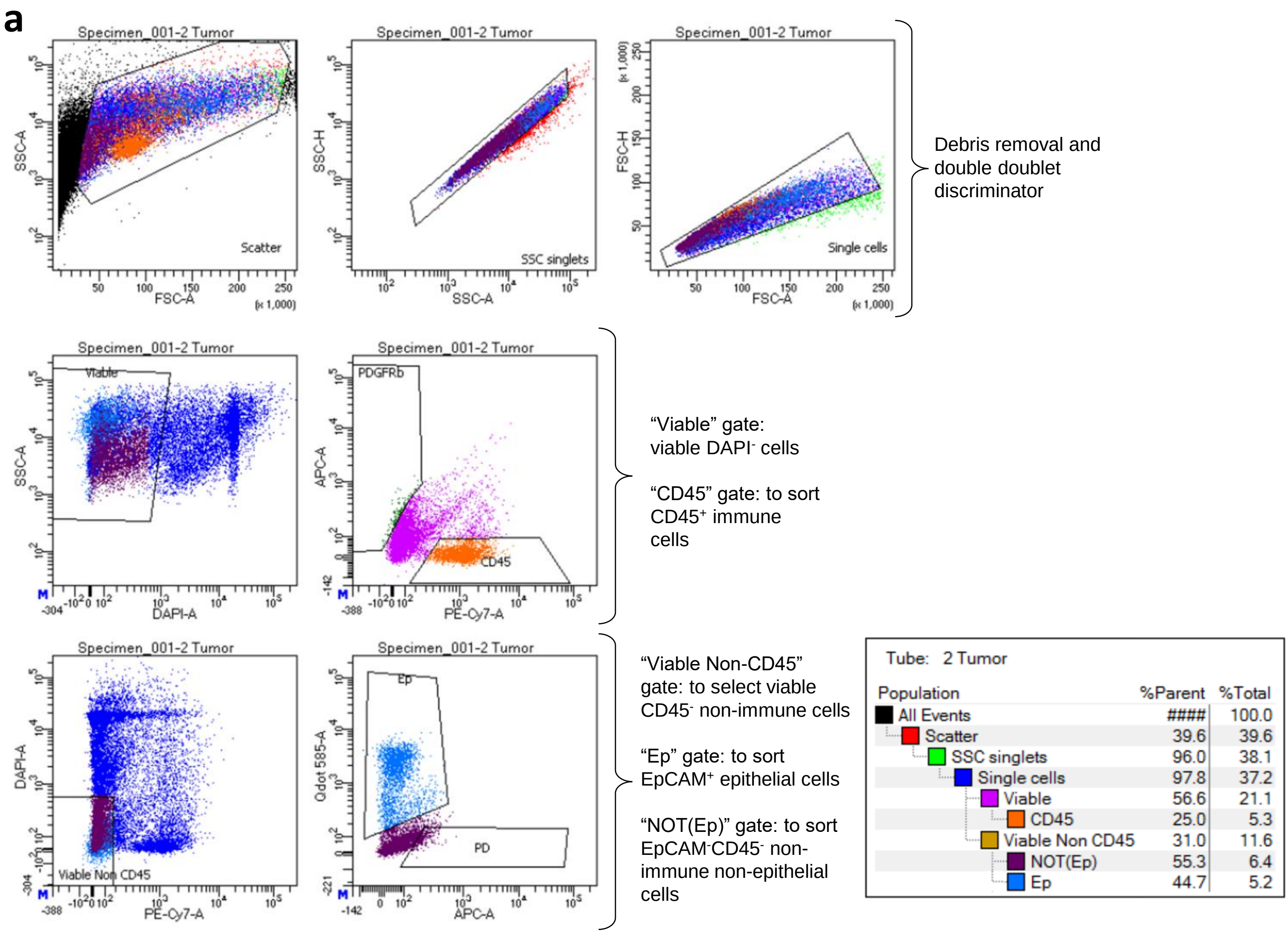

b

Summary of Quality Metrics

Sample ID	Count	Cell	Gene	R-Count	R-Gene	mtRNA	rRNA
ICC1	99546488	8689	36601	[500-7158-104972]	[17-1344-7171]	99.40%	52.90%
ICC2	41447821	5603	36601	[500-4574-67558]	[19-1623-7198]	99.50%	53.80%
ICC3	79655392	10636	33538	[500-4396-94965]	[55-1618-8023]	95.80%	57.20%
ICC4	81770180	9821	36601	[500-3635-89980]	[40-1323-7676]	98.40%	53.20%
ICC5	88015520	13011	36601	[500-3941-84100]	[30-1420-7842]	99.10%	65.70%
ICC6	118576967	16581	36601	[500-4005-111520]	[53-1571-9505]	95.10%	50.40%
ICC7	70731952	9019	36601	[500-4926-92069]	[34-1793-9125]	97.90%	39.30%

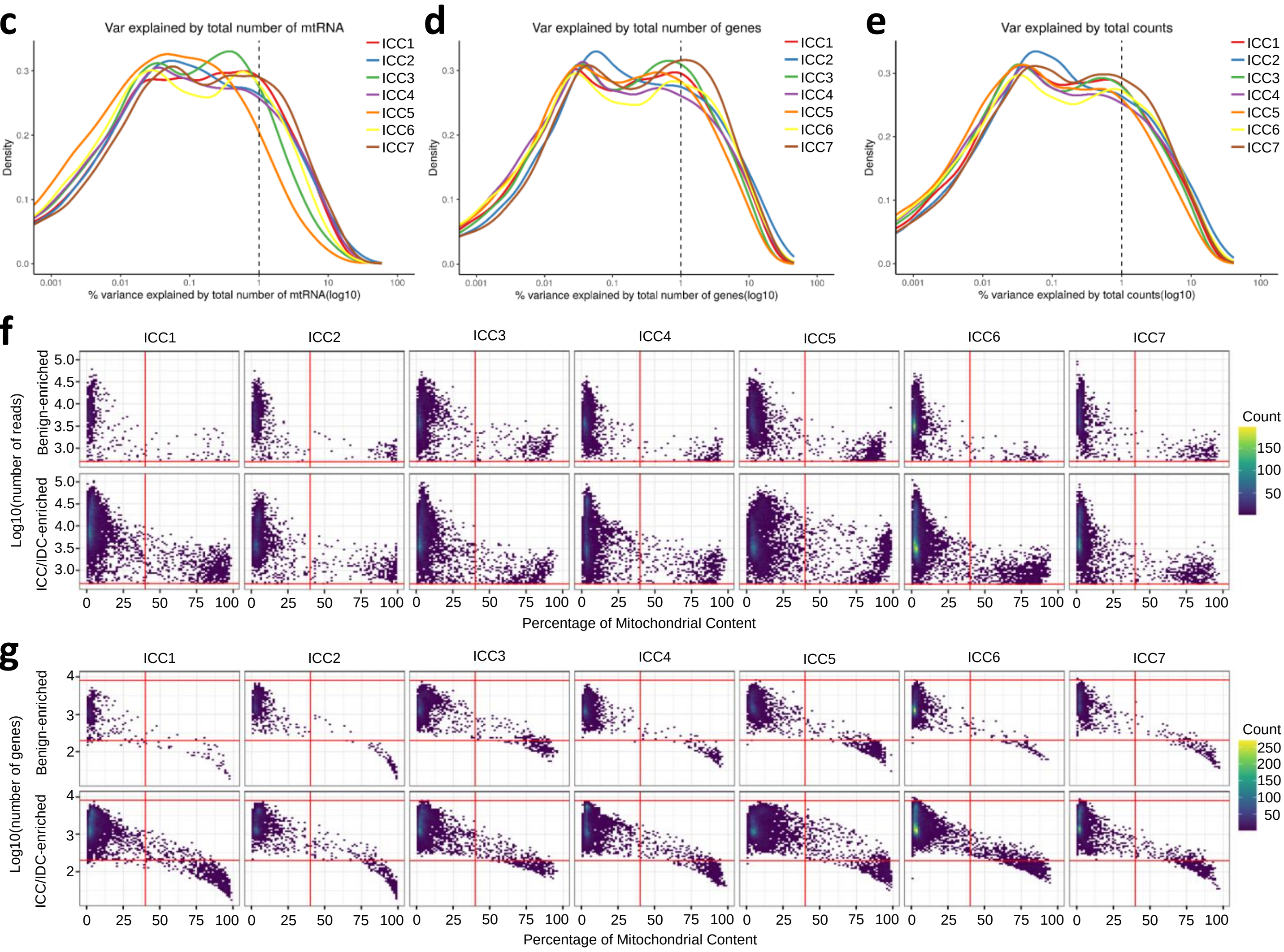

Supplementary Figure 10. Fluorescence-activated cell sort gating and scRNAseq quality metrics. a. Representative gating for sorting by flow cytometry. **b.** Summary of quality metrics for raw data: Count, total read count; Cell, total cell count; Gene, total gene count; R-Count, range of total number of read counts per cell; R-Gene, range of total number of genes per cell; mtRNA, maximum percentage of mitochondrial content; rRNA, maximum percentage of ribosomal content. **c-e.** Density plots of the percentage of expression variances explained by log-transformed mitochondrial content (c), total number of genes (d), and total number of read counts (e) indicate minimal technical variance and batch effect between samples. **f, g.** Scatter plots of log-transformed number of reads (f) or number of genes (g) versus percentage of mitochondrial content. Vertical red line indicates mitochondrial content of 40% (f, g); horizontal red lines indicate 500 reads (f), or 200 and 8,000 genes (g).

Supplementary Table 1. Patient Characteristics (n=7)	
**Mean Age (years; range)**	65 (52-74)
**Race**	
African American/Black	1
European American/White	6
**Mean Pre-biopsy PSA (ng/mL; range)**	10.0 (3.1-18.1)
**Biopsy Grade Group**	
Grade Group 2 (Gleason score 3+4=7)	3
Grade Group 3 (Gleason score 4+3=7)	4
**Pathologic Grade Group**	
Grade Group 2 (Gleason score 3+4=7)	2
Grade Group 3 (Gleason score 4+3=7)	4
Grade Group 5 (Gleason score 4+5=9)	1
**Cribriform (ICC)**	7
**IDC**	6
**Pathologic stage (AJCC TNM 8th Edition)**	
pT3aNX	2
pT3aN0	4
pT3bN0	1
**Surgical Margin**	
Negative	5
Positive	2

Supplementary Table 2. Number of Cells Analyzed per Patient after Filtering

Sample	Total Cells	ICC/IDC-enriched	Benign-enriched
**ICC1**	7,080	6,359	721
**ICC2**	4,716	3,371	1,345
**ICC3**	8,431	5,717	2,714
**ICC4**	7,394	4,931	2,463
**ICC5**	9,576	6,446	3,130
**ICC6**	13,431	9,470	3,961
**ICC7**	7,069	5,111	1,958
**Total Cells**	57,697	41,405	16,292
**Mean Cells**	8,242	5,915	2,327

Supplementary Table 3. Percent of Cells Analyzed per Patient

Sample	% ICC/IDC-enriched	% Benign-enriched
**ICC1**	89.8	10.2
**ICC2**	71.5	28.5
**ICC3**	67.8	32.2
**ICC4**	66.7	33.3
**ICC5**	67.3	32.7
**ICC6**	70.5	29.5
**ICC7**	72.3	27.7
**Mean %**	72.3	27.7

Supplementary Table 4. Number of VDJ Cells Analyzed per Patient after Filtering

Sample	Total Cells	VDJ Cells	VDJ Cells in ICC/IDC-enriched	VDJ Cells in Benign-enriched
**ICC1**	7,080	1,796	1,543	253
**ICC2**	4,716	1,468	1,092	376
**ICC3**	8,431	1,878	1,254	624
**ICC4**	7,394	1,920	862	1,058
**ICC5**	9,576	2,941	1,839	1,102
**ICC6**	13,431	3,608	2,615	993
**ICC7**	7,069	2,059	1,610	449
**Total Cells**	57,697	15,670	10,815	4,855
**Mean Cells**	8,242	2,239	1,545	694

Supplementary Table 5. Percent of VDJ Cells Analyzed per Patient

Sample	VDJ %	VDJ % in ICC/IDC-enriched	VDJ % in Benign-enriched
**ICC1**	25.4	24.3	35.1
**ICC2**	31.1	32.4	28
**ICC3**	22.3	21.9	23
**ICC4**	26	17.5	43
**ICC5**	30.7	28.5	35.2
**ICC6**	26.9	27.6	25.1
**ICC7**	29.1	31.5	22.9
**Mean %**	27.3	26.2	30.3

Supplementary Table 6. Number of Cells Analyzed per Cell Category

Region	Total Cells	Epithelial	Immune TME	Non-immune TME
**ICC/IDC-enriched**	41,405	14,668	20,464	6,273
**Benign-enriched**	16,292	3,073	9,719	3,500
**Total**	57,697	17,741	30,183	9,773

Supplementary Table 7. Number of Cells Analyzed per Cluster

Cluster	Total	ICC/IDC-enriched	Benign-enriched
**0**	6,441	4,197	2,244
**1**	5,568	4,148	1,420
**2**	4,218	3,225	993
**3**	4,050	2,552	1,498
**4**	3,696	2,660	1,036
**5**	3,675	3,528	147
**6**	2,978	2,923	55
**7**	2,921	1,847	1,074
**8**	2,838	1,965	873
**9**	2,220	1,773	447
**10**	2,156	1,592	564
**11**	2,031	1,998	33
**12**	1,956	928	1,028
**13**	1,864	1,164	700
**14**	1,714	1,569	145
**15**	1,533	998	535
**16**	1,439	63	1376
**17**	1,433	939	494
**18**	1,424	1,037	387
**19**	884	625	259
**20**	749	408	341
**21**	673	431	242
**22**	590	458	132
**23**	463	288	175
**24**	129	46	83
**25**	54	43	11
**Total**	57,697	41,405	16,292

Supplementary Table 8. Key Resources

REAGENT or RESOURCE	SOURCE	IDENTIFIER
**Antibodies for IHC**	**Source**	**Identifier**
PTEN	DAKO	M3627 RRID:AB_2174185
CD31	Novacastra	CD31-607-L-CE
ERG	Biocare Medical	PM421AA RRID:AB_10804416
Pros C	Biocare Medical	API3154DSAA
AR	Roche	760-4605 SP107
MYC	Abcam	ab32072 RRID:AB_731658
**Probes for RNAscope**		
SCHLAP1	ACD	534271
JAG1	ACD	546181-C2
CTHRC1	ACD	413331
ASPN	ACD	404481
FAP	ACD	411971
ENG	ACD	484111
KRT13	ACD	843401
SCGB1A1	ACD	469971-C2
**Fluorescence Activated Cell Sorting (FACS)**		
Human TruStain FcX™ (Fc Receptor Blocking Solution)	BioLegend	422302 RRID:AB_2818986
DAPI (4',6-Diamidino-2-Phenylindole, Dilactate)	Invitrogen	D3571
APC anti-human CD140b (PDGFRβ) Antibody (Clone 18A2)	BioLegend	323608 RRID:AB_2162787
Brilliant Violet 711™ anti-human CD326 (Ep-CAM) Antibody (Clone 9C4)	BioLegend	324239 RRID:AB_2734306
PE/Cy7 anti-human CD45 Antibody (Clone HI30)	BioLegend	304015 RRID:AB_314403
**Antibodies for Feature Barcoding**		
TotalSeq™-C0251 anti-human Hashtag 1 Antibody (Clones LNH-94; 2M2)	BioLegend	394661 RRID:AB_2801031
TotalSeq™-C0252 anti-human Hashtag 2 Antibody (Clones LNH-94; 2M2)	BioLegend	394663 RRID:AB_2801032
PE/Cyanine7 anti-human CD298 Antibody (Clone LNH-94)	BioLegend	341707 RRID:AB_2819970
PE/Cy7 anti-human β2-microglobulin Antibody (Clone 2M2)	BioLegend	316317 RRID:AB_2632830
**Tissue Dissociation**		
Tumor Dissociation Kit (human)	Miltenyi Biotec	130-095-929
MACS SmartStrainers (70 µm)	Miltenyi Biotec	130-098-462
gentleMACS C Tubes	Miltenyi Biotec	130-093-237
gentleMACS Octo Dissociator with Heaters	Miltenyi Biotec	130-096-427
Red Blood Cell Lysis Solution (10×)	Miltenyi Biotec	130-094-183
RPMI 1640 Medium, no phenol red	Gibco	11835030
Fetal Bovine Serum, qualified, United States	Gibco	26140079
Countess Cell Counting Chamber	Invitrogen	C10283
**scRNA-seq Library Construction**		
v1: Chromium Single Cell V(D)J Reagent Kits with Feature Barcode technology for Cell Surface Protein	10X Genomics	User Guide CG000186 Rev D
v1: Chromium Single Cell 5' Library & Gel Bead Kit, 16 rxns	10X Genomics	PN-1000006
v1: Chromium Single Cell 5' Library Construction Kit, 16 rxns	10X Genomics	PN-1000020
v1: Chromium Single Cell 5' Feature Barcode Library Kit, 16 rxns	10X Genomics	PN-1000080
v1: Chromium Single Cell V(D)J Enrichment Kit, Human T Cell, 96 rxns	10X Genomics	PN-1000005
v1: Chromium Single Cell A Chip Kit, 16 rxns	10X Genomics	PN-1000151
v1: Chromium i7 Multiplex Kit, 96 rxns	10X Genomics	PN-120262
v1: Chromium i7 Multiplex Kit N, Set A, 96 rxn	10X Genomics	PN-1000084
v2: Chromium Next GEM Single Cell 5' Reagent Kits v2 (Dual Index) with Feature Barcode technology for Cell Surface Protein & Immune Receptor Mapping	10X Genomics	User Guide CG000330 Rev A
v2: Chromium Next GEM Single Cell 5' Kit v2, 4 rxns	10X Genomics	PN-1000265
v2: Library Construction Kit, 16 rxns	10X Genomics	PN-1000190
v2: 5' Feature Barcode Kit, 16 rxns	10X Genomics	PN-1000256
v2: Chromium Single Cell Human TCR Amplification Kit, 16 rxns	10X Genomics	PN-1000252
v2: Chromium Next GEM Chip K Single Cell Kit, 16 rxns	10X Genomics	PN-1000287
v2: Dual Index Kit TT Set A, 96 rxns	10X Genomics	PN-1000215
v2: Dual Index Kit TN Set A, 96 rxns	10X Genomics	PN-1000250
**Next Generation Sequencing**		
NovaSeq 6000	Illumina	
**Software and Algorithms**		
Cell Ranger 5.0.0	10X Genomics	
R software	https://www.r-project.org/	
scRNABatchQC	https://github.com/liuqivandy/scRNABatchQC	
Seurat	https://satijalab.org/seurat/	
edgeR	https://bioconductor.org/packages/release/bioc/html/edgeR.html	
GSEA	https://www.gsea-msigdb.org/gsea/index.jsp	
Monocle	http://cole-trapnell-lab.github.io/monocle-release/	
QuPath v0.3.0	https://qupath.github.io	
GraphPad Prism 9		
Excel		
Procreate		
**Sequencing Data**		
scRNA-seq data	GEO	Series GSE185344
**Code Availability**		
Code	GitHub	https://github.com/shengqh/Hurley2022scRNA/.
