# Supplementary material for: Single cell analysis of cribriform prostate cancer reveals cell intrinsic and tumor microenvironmental pathways of aggressive disease
Source: Nat Commun. 2022 Oct 13;13:6036. doi: 10.1038/s41467-022-33780-1 (PMC9562361; doi:10.1038/s41467-022-33780-1)
Supplement: Supplementary file 2 — Reporting Summary [file 41467_2022_33780_MOESM2_ESM.pdf]

nature portfolio

Corresponding author(s): Paula Hurley

Last updated by author(s): 9/10/2022

Reporting Summary

Nature Portfolio wishes to improve the reproducibility of the work that we publish. This form provides structure for consistency and transparency in reporting. For further information on Nature Portfolio policies, see our Editorial Policies and the Editorial Policy Checklist.

Statistics

For all statistical analyses, confirm that the following items are present in the figure legend, table legend, main text, or Methods section.

n/a	Confirmed	
☐	☒	The exact sample size (*n*) for each experimental group/condition, given as a discrete number and unit of measurement
☐	☒	A statement on whether measurements were taken from distinct samples or whether the same sample was measured repeatedly
☐	☒	The statistical test(s) used AND whether they are one- or two-sided<br>*Only common tests should be described solely by name; describe more complex techniques in the Methods section.*
☐	☒	A description of all covariates tested
☐	☒	A description of any assumptions or corrections, such as tests of normality and adjustment for multiple comparisons
☐	☒	A full description of the statistical parameters including central tendency (e.g. means) or other basic estimates (e.g. regression coefficient) AND variation (e.g. standard deviation) or associated estimates of uncertainty (e.g. confidence intervals)
☐	☒	For null hypothesis testing, the test statistic (e.g. *F*, *t*, *r*) with confidence intervals, effect sizes, degrees of freedom and *P* value noted<br>*Give P values as exact values whenever suitable.*
☒	☐	For Bayesian analysis, information on the choice of priors and Markov chain Monte Carlo settings
☒	☐	For hierarchical and complex designs, identification of the appropriate level for tests and full reporting of outcomes
☒	☐	Estimates of effect sizes (e.g. Cohen's *d*, Pearson's *r*), indicating how they were calculated

Our web collection on statistics for biologists contains articles on many of the points above.

Software and code

Policy information about availability of computer code

Data collection	For scRNA-seq, libraries were sequenced with the NovaSeq 6000 and raw data were collated as paired FASTQ reads for hashtags (feature barcoding), 5' transcriptome, and VDJ (TCR-seq) sequences.
Data analysis	Code can be found on GitHub: https://github.com/shengqh/Hurley2022scRNA/.<br><br>For scRNA-seq<br>10x Genomics Cell Ranger v5.0.0 was used to build reference genome index, map reads to reference genome (GRCh38-2020-A) and quantify genes. Sample-specific hashtags for were demultiplexed by in-house scripts. Whole transcriptome data and hashtag data were stored in the RNA and ADT (Antibody-Derived Tags) assay slots of a S4 Seurat object (RDS format). R v4.1.0 was used for downstream R analyses. scRNABatchQC v0.10.4 was used to verify consistency and minimal variance of sequenced data based on quality metrics such as unique gene and cell counts. Seurat v4.1.0 was used for clustering analysis with sctransform based normalization. Cell type of each cluster was initially classified based on cell activity database and then manually refined based on cell type specific marker gene expression. edgeR v3.36.0 was used to detect differential expression across conditions. Modified GSEA v4.2.1 was used for paired pathway analysis. Monocle3 v1.0.1 package was used for pseudotime analyses. 10x Genomics Cell Ranger v5.0.0 and enclone v0.5.10 were used for VDJ TCR analysis. Simpson clonality (square root of the Simpson's index, which is the summation of the square of proportional abundance of all observed clonotypes) was used to determine T cell clonotype evenness. R packages ggplot2 v3.3.5 and reshape2 v1.4.4 were used to graph dimplots, featureplots, and violin plots; R packages tidyverse v1.3.1 and pheatmap v1.0.12 were used to plot heatmaps; Reclustering was performed with R packages patchwork v1.1.1, kableExtra v1.3.4, and dplyr v2.1.1.<br><br>For survival analysis<br>R v4.1.0 was used for downstream R analyses. Core survival analysis based on collated Z-scores was performed using R package survival v3.2-13, and Kaplan-Meier survival curves were plotted with R packages survminer v0.4.9 and ggplot2 v3.3.5.

Statistical comparisons were performed using GraphPad Prism software (v5.0) or Seurat V3 in R Studio.

FACS Analysis
BD FACS Diva (v8.0.1) software was used to analyze FACS data.

For manuscripts utilizing custom algorithms or software that are central to the research but not yet described in published literature, software must be made available to editors and reviewers. We strongly encourage code deposition in a community repository (e.g. GitHub). See the Nature Portfolio guidelines for submitting code & software for further information.

Data

Policy information about availability of data

All manuscripts must include a data availability statement. This statement should provide the following information, where applicable:

- Accession codes, unique identifiers, or web links for publicly available datasets
- A description of any restrictions on data availability
- For clinical datasets or third party data, please ensure that the statement adheres to our policy

The publicly available Prostate Adenocarcinoma (TCGA, PanCancer Atlas) data and MSKCC Prostate Adenocarcinoma data are available from cBioPortal https://www.cbioportal.org/datasets. The RIKEN FANTOM5 database is publicly available: https://fantom.gsc.riken.jp/5/. The single-cell RNA-sequencing data generated in this study have been deposited in the Gene Expression Omnibus (GEO) database under accession code GSE185344: https://www.ncbi.nlm.nih.gov/geo/query/acc.cgi?acc=GSE185344. Source data are provided in this paper as a Source data file. The remaining data are available within the Article, Supplementary Information, and Source Data file.

Field-specific reporting

Please select the one below that is the best fit for your research. If you are not sure, read the appropriate sections before making your selection.

☒ Life sciences ☐ Behavioural & social sciences ☐ Ecological, evolutionary & environmental sciences

For a reference copy of the document with all sections, see nature.com/documents/nr-reporting-summary-flat.pdf

Life sciences study design

All studies must disclose on these points even when the disclosure is negative.

Sample size	Patient sample size for single-cell RNA sequencing was based on pilot experiments of human prostate cancer single-cell dissociation followed by flow cytometry, target of 5,000 or more cells per patient, and prior data generated by our group examining gene expression by RNAscope in fibroblasts adjacent to benign prostate glands and in cancer associated fibroblasts (CAF) adjacent to ICC/IDC foci. Pilot experiments analyzed single-cell dissociated human prostate tissue from RP by flow cytometry for several subtypes of live (DAPI-) cells: epithelial cells (EPCAM+), immune cells (CD45+), fibroblasts (PDGFRB+), and other cells (EPCAM-CD45-PDGFRB-). These experiments indicated that PDGRRB+ cells were low abundant compared to the other cell types analyzed. To power our study for low abundant cell types, we performed sample size power calculations using two fibroblast makers (ASPN and FAP) that were differentially expressed in ICC/IDC CAF compared to fibroblasts adjacent to benign prostate glands by RNAscope. ASPN expression by RNAscope in benign-adjacent fibroblasts (17.43±11.20; n=21) and ICC-adjacent fibroblasts (144.5±56.21; n=12) yielded an effect size d=3.13 while FAP expression by RNAscope in benign-adjacent fibroblasts (2.75 ±2.24; n=11) and ICC-adjacent fibroblasts (71.0±48.3; n=6) resulted in an effect size d=2.00. Using a two-sided test with α error of probability = 0.05, power = 0.8, and an allocation ratio of control/experimental=1, the sample size should be 3 and 6 patients, respectively to demonstrate a statistically significant difference between paired benign and ICC/IDC prostate tissue. To account for error, we examined paired benign and ICC/IDC prostate tissue from 7 patients. We acknowledge that these power calculations were not specifically tailored to single-cell RNA sequencing of thousands of cells per patient, but we reasoned that they were a conservative estimate of the patient sample size needed for statistical power. Ultimately, this study examined over 50,000 cells in total coming from paired ICC/IDC-enriched and benign-enriched regions from 7 patients.
Data exclusions	Quality control was performed on single-cell RNA sequencing data, and it is described in the Methods section under Data Analysis and shown in Supplemental Figure S10. Manufacturer recommendation, prior studies, and analysis of our samples were considered prior to defining data exclusion criteria. Double positive and double negative cells with both or none of ICC/IDC-enriched and Benign-enriched hashtags were removed. Low quality cells as determined by mitochondrial content, unique reads, and unique read counts were also removed from analyses.
Replication	This study included paired ICC/IDC-enriched and benign-enriched tissue from 7 patients (biological replicates). For all patients, fresh samples were used for single-cell RNA sequencing, and thus technical replicates of single-cell RNA sequencing were not feasible. However, key findings from single-cell RNA sequencing were validated using RNAscope or IHC in FFPE tissue. Seven additional patients were enrolled but excluded due to prostate volume at RP below threshold for research sampling or inability to locate adequate ICC/IDC for scRNAseq. Samples were not isolated from the excluded patients. All staining for validation/replication was reported in the manuscript. Pathway analyses also considered patient pairs.
Randomization	This study did not involve randomization. Prostate tissue was assigned according to the pathology status, either benign-enriched or ICC/IDC-enriched.
Blinding	Blinding was not relevant to this study. Prostate tissue was assigned according to the pathology status, either benign-enriched or ICC/IDC-enriched.

Reporting for specific materials, systems and methods

We require information from authors about some types of materials, experimental systems and methods used in many studies. Here, indicate whether each material, system or method listed is relevant to your study. If you are not sure if a list item applies to your research, read the appropriate section before selecting a response.

Materials & experimental systems

n/a	Involved in the study	
☐	☒	Antibodies
☒	☐	Eukaryotic cell lines
☒	☐	Palaeontology and archaeology
☒	☐	Animals and other organisms
☐	☒	Human research participants
☒	☐	Clinical data
☒	☐	Dual use research of concern

Methods

n/a	Involved in the study	
☒	☐	ChIP-seq
☐	☒	Flow cytometry
☒	☐	MRI-based neuroimaging

Antibodies

Antibodies used

Antibodies for Fluorescence Activated Cell Sorting (FACS):
APC anti-human CD140b (PDGFRβ) Antibody (BioLegend, Cat# 323608, RRID:AB_2162787, Clone 18A2, Lot# B285844, dilution 1:80);
Brilliant Violet 711 anti-human CD326 (Ep-CAM) Antibody (BioLegend, Cat# 324239, RRID:AB_2734306, Clone 9C4, Lot# B259479, dilution 1:2500);
PE/Cy7 anti-human CD45 Antibody (BioLegend, Cat# 304015, RRID:AB_314403, Clone HI30, Lot# B264587, dilution 1:5000).

Antibodies for Feature Barcoding:
TotalSeq-C0251 anti-human Hashtag 1 Antibody (BioLegend, Cat# 394661, RRID:AB_2801031, Clones LNH-94 and 2M2, Lot# B296354, 0.15ug antibody per 100ul staining volume);
TotalSeq-C0252 anti-human Hashtag 2 Antibody (BioLegend, Cat# 394663, RRID:AB_2801032, Clones LNH-94 and 2M2, Lot# B294923, 0.15ug antibody per 100ul staining volume);
PE/Cyanine7 anti-human CD298 Antibody (BioLegend, Cat# 341707, RRID:AB_2819970, Clone LNH-94, Lot# B297300, 0.15ug antibody per 100ul staining volume);
PE/Cyanine7 anti-human β2-microglobulin Antibody (BioLegend, Cat# 316317, RRID:AB_2632830, Clone 2M2, Lot# B264876, 0.15ug antibody per 100ul staining volume).

Antibodies for Immunohistochemistry (IHC)
PTEN (DAKO, RRID:AB_2174185, Cat# M3627, dilution 1:250)
ERG (Biocare Medical, RRID:AB_10804416, Cat# PM421AA, dilution Ready to Use)
ProsC (Biocare Medical, Cat# API3154DSSAA, dilution Ready to Use)
CD31 (Leica; Cat# PA0250, dilution Ready to Use)
AR (Roche, Cat# 760-4605 SP107, dilution Ready to Use)
MYC (Abcam, Cat# ab32072, dilution 1:100)

Validation

FACS antibodies were titrated using freshly dissociated human prostate tumor cells and optimized at respective dilutions shown above.

Nucleotide-linked hashtag antibodies (TotalSeq-C0251 and TotalSeq-C0252) were titrated based on PE/Cyanine7-tagged versions of antibodies against human CD298 and β2-microglobulin using freshly dissociated human prostate tumor cells and were optimized at 0.15ug per 100ul of staining volume.

IHC antibodies for ERG and ProsC were previously validated for clinical use by the VUMC clinical pathology laboratory. Antibodies were validated on 10 known human positive cases and 10 known human negative cases. The medical director reviewed and confirmed the accuracy of the antibody staining. Experimental IHC slides had control tissue included.

IHC antibodies for PTEN, CD31, AR, and MYC were previously validated for research use by the VUMC translational pathology shared resource using known human positive and negative control tissue. Experimental IHC slides had control slides included. The study pathologist confirmed the accuracy of the antibody staining.

Human research participants

Policy information about studies involving human research participants

Population characteristics	All research participants were male with a mean age of 65 years old, which is consistent with sex and age demographics for prostate cancer. Full population characteristics can be found in Supplementary Table 1. All research participants had a diagnosis of prostate cancer, did not have prior treatment for prostate cancer, and were undergoing a radical prostatectomy (RP) for the treatment of prostate cancer.
Recruitment	This study protocol was approved by the Vanderbilt University Medical Center (VUMC) Institutional Review Board (IRB) (Nashville, TN). Written informed consent was obtained for all patients prior to enrollment by the Cooperative Human Tissue Network at VUMC. Patients were not compensated for participation. This study adhered to the Declaration of Helsinki principles. Enrolled patients were excluded if their prostate volume at RP was below the threshold allowed for research sampling or due to an inability to locate adequate ICC/IDC for analyses. There were no other self-selection bias or other biases that would impact these results.
Ethics oversight	The Institutional Review Board (IRB) of Vanderbilt University Medical Center approved the study protocol.

Note that full information on the approval of the study protocol must also be provided in the manuscript.

Flow Cytometry

Plots

Confirm that:

- [x] The axis labels state the marker and fluorochrome used (e.g. CD4-FITC).
- [x] The axis scales are clearly visible. Include numbers along axes only for bottom left plot of group (a 'group' is an analysis of identical markers).
- [x] All plots are contour plots with outliers or pseudocolor plots.
- [x] A numerical value for number of cells or percentage (with statistics) is provided.

Methodology

Sample preparation	Prostate tissue dissociation<br>Human prostate tissues from radical prostatectomy were dissociated mechanically and enzymatically into single-cell suspensions with the Tumor Dissociation Kit (human, Miltenyi Biotec) as per manufacturer's protocol. Briefly, tissues were cut into 1-2 mm pieces and then transferred into a gentleMACS C Tube for dissociation on the gentleMACS Octo Dissociator with Heaters using the m_imptumor_01 and 37C_h_TDK2 programs. Dissociated cell suspension was passed through a 70 µm MACS SmartStrainer and cells were harvested by spinning twice at 300 x g for 7min. Erythrocytes were removed with Red Blood Cell Lysis Solution (10x) as per manufacturer's protocol. Cell number and viability were evaluated with the Invitrogen Countess Automated Cell Counter.<br><br>Fluorescence Activated Cell Sorting (FACS)<br>Dissociated cells were resuspended in FACS buffer (5% FBS in PBS) and Fc blocked for 10min. Cells were stained in the dark for 30 minutes at room temperature with APC-PDGFRβ (1:80), BV711-Ep-CAM (1:2500), PE/Cy7-CD45 (1:5000) and washed 3 times with FACS buffer. For every wash step, cells were centrifuged at 300g for 5 minutes at 4 degrees Celsius. DAPI (1:100) was then added to stain dead cells. Cells were kept in the dark at room temperature for at least 15 minutes, then kept on ice while setting up cytometer for sorting. When cell yields were below threshold for sorting (samples for patients ICC3 and benign-enriched tissue of patients ICC1 and ICC6), only DAPI (1:100) was added to sort for viable cells. Sorting was performed at a flow rate of 1.0, with >90% consistent efficiency.
Instrument	BD FACSAria™ III Cell Sorter in a Baker BioPROTECT® IV Baker Hood Enclosure
Software	BD FACS Diva 8.0.1
Cell population abundance	Viable (DAPI-) cells were sorted into three populations: immune (CD45+), epithelial (EpCAM+), and other (non-immune tumor microenvironment cells, CD45-Ep-CAM-) cells. Cell population abundance was patient-dependent. Overall, we yielded an average of 51,385 epithelial, 66,231 immune, and 93,229 other viable cells from sorting an average starting number of 856,000 dissociated cells. For specimens from patient ICC3 and benign-enriched tissue of patients ICC1 and ICC6, we yielded an average of 46,269 viable cells from sorting an average starting number of 123,000 dissociated cells. To ensure maximum cell input for scRNA-seq, purity was determined by observing consistent morphology in debris-free cell populations with light microscopy (4X to 40X photographs taken with EVOS XL Core).

Gating strategy

As shown in our representative gating strategy in Figure S10a, a main gate (SSC-A vs FSC-A) was first drawn to exclude debris and aggregates, followed by two consecutive doublet discriminator gates (SSC-H vs SSC-A and FSC-H vs FSC-A) to select single cells. Viable single cells (DAPI-) were selected by the “Viable” gate (SSC-A vs DAPI-A). Viable immune cells (CD45+) were then selected by the “CD45” gate (APC-A vs PE-Cy7-A). To select epithelial (Ep-CAM+) and the other (non-immune tumor microenvironment cells, CD45-Ep-CAM-) cells, a “Viable Non-CD45” gate (DAPI-A vs PE-Cy7-A) was first drawn, from which the epithelial cells were selected in the “Ep” gate and the other cells were selected in the “NOT(Ep)” gate (Qdot 585(BV711)-A vs APC-A). Percentages were shown in Figure S10a.

☒ Tick this box to confirm that a figure exemplifying the gating strategy is provided in the Supplementary Information.
